# Comparative Neutralization Activity of Commercial Rabies Immunoglobulin against Diverse Lyssaviruses

**DOI:** 10.3390/vaccines11071255

**Published:** 2023-07-18

**Authors:** Jessica Coertse, Natalie Viljoen, Jacqueline Weyer, Wanda Markotter

**Affiliations:** 1 Centre for Emerging Zoonotic and Parasitic Diseases, National Institute for Communicable Diseases, A Division of the National Health Laboratory Service, Johannesburg 2131, South Africa; jessicac@nicd.ac.za (J.C.); nataliev@nicd.ac.za (N.V.); jacquelinew@nicd.ac.za (J.W.); 2 Centre for Viral Zoonoses, Department of Medical Virology, Faculty of Health Sciences, University of Pretoria, Pretoria 0001, South Africa; 3 Department of Microbiology and Infectious Diseases, School of Pathology, University of Witwatersrand, Johannesburg 2131, South Africa

**Keywords:** lyssavirus, post-exposure prophylaxis, rabies immunoglobulin, passive immunization, rabies

## Abstract

Novel lyssaviruses, the causative agents of rabies, continue to be described mostly due to increased surveillance in bat hosts. Biologicals for the prevention of rabies in humans have, however, remained largely unchanged for decades. This study aimed to determine if commercial rabies immunoglobulin (RIG) could neutralize diverse lyssaviruses. Two commercial preparations, of human or equine origin, were evaluated against a panel consisting of 13 lyssavirus species. Reduced neutralization was observed for the majority of lyssaviruses compared to rabies virus and was more evident for lyssaviruses outside of phylogroup I. Neutralization of more diverse lyssaviruses only occurred at very high doses, except for Ikoma lyssavirus, which could not be neutralized by the RIG evaluated in this study. The use of RIG is a crucial component of rabies post-exposure prophylaxis and the data generated here indicate that RIG, in its current form, will not protect against all lyssaviruses. In addition, higher doses of RIG may be required for neutralization as the genetic distance from vaccine strains increases. Given the limitations of current RIG preparations, alternative passive immunization options should be investigated.

## 1. Introduction

Rabies is an acute fatal encephalomyelitis caused by members of the expanding *Lyssavirus* genus. At the time of this report, the genus included 17 species, i.e., *Lyssavirus aravan*, -*autralis*, -*bokeloh*, -*caucasicus*, -*duvenhage*, -*formosa*, -*gannoruwa*, -*hamburg*, -*helsinki*, -*ikoma*, -*irkut*, -*khujand*, -*lagos*, -*lleida*, -*mokola*, -*rabies* and -*shimoni* with one unclassified virus, i.e., Kotalahti bat lyssavirus (KBLV) [1] and two potentially novel species, Matlo bat lyssavirus (MBLV) [2] and Taiwan bat lyssavirus 2 (TWBLV-2) [3]. Rabies is one of the deadliest known viral infections and is reported in more than 150 countries and territories, causing tens of thousands of human deaths annually [4,5]. Following exposure to a potentially rabid animal, prompt post-exposure prophylaxis (PEP) is recommended consisting of thorough washing and flushing of the wound, the initiation of a series of rabies vaccines, and, for category III exposures, the infiltration of rabies immunoglobulin (RIG) into and around all wounds [6]. Vaccines for rabies prevention were first used successfully in 1885 by Pasteur and several improvements followed over subsequent decades. However, several adverse effects were reported for nerve tissue, chicken embryo-, and duck embryo-cell vaccines, which have mostly been replaced with vaccines produced in Vero cells since the 1980s that are not only safer but also more potent [4,7]. Only three rabies virus (RABV) strains have been well established for human rabies vaccine manufacturing, i.e., the Louis Pasteur (PAS) virus and derivatives, the Flury virus and derivatives, and the Street Alabama Dufferin (SAD) virus and derivatives [7]. The efficacy of passive immunization using RIG was first illustrated in the 1950s [8] and was included in the World Health Organization (WHO) guidelines for rabies PEP soon thereafter [9]. Initial studies indicated that the administration of RIG could interfere with immunity induced by the rabies vaccine, and it was therefore recommended that RIG should be infiltrated into wounds or given intramuscularly (i.m.) at a dose of 0.5 mL/kg body weight, followed by 14 daily doses of vaccine and 1–3 booster vaccinations [9]. To overcome uncertainties in RIG dosage, a study was undertaken to determine the maximum dose that could be administered without inhibition of the vaccine-induced immune response [10], and the WHO guidelines were subsequently amended to specify that 20 IU/kg body weight of human rabies immunoglobulin (hRIG) or 40 IU/kg body weight of heterologous serum should be administered as a single dose i.m. post-exposure [11,12]. Shortly thereafter, guidelines were changed to indicate that the complete RIG dose should be infiltrated into any wounds if anatomically feasible and any remaining RIG should be given i.m. [13]. Other than a recent change to the guidelines, which stated that RIG should only be infiltrated into and around wounds and not be administered i.m. at sites distant from the wound sites, the guidelines have remained unchanged [6]. Since the 1950s, the WHO has acknowledged that immunity can only be estimated by the presence of neutralizing antibodies [9] and post-exposure treatment guidelines were therefore based on the level of circulating neutralizing antibodies. Importantly, this approach does not consider the size and number of wounds or the local neutralizing effect of RIG. Since the first rabies vaccine in the late 19th century, several improvements have been made with regard to purity, safety, and immunogenicity. Current vaccine schedules have also been reduced from over 14 doses to only a handful of doses; however, RIG treatment guidelines have remained largely unchanged for the last five decades. The purpose of the administration of RIG is to neutralize any virus present in the wound to reduce the viral load before the virus can replicate or enter the peripheral nerve endings and to stimulate a T-lymphocyte-mediated immune response. This provides some level of protection before the appearance of vaccine-induced neutralizing antibodies 1–2 weeks after vaccination [12,13,14,15]. All previous dose determinations of RIG treatment were based on the level of circulating antibodies before and after vaccination; however, a previous study has indicated that RIG administered i.m. does not provide effective systemic protection against infection [15], i.e., provision of antibodies at the wound site is much more effective and the level of circulating antibodies less important. Some of the most commonly encountered causes of rabies PEP treatment failure involve the absence or incorrect use of RIG [16,17,18]. This emphasizes the importance of RIG in treatment; however, RIG is extremely expensive, and is in short supply due to limited production [19,20]. This scarce and essential biological product should then be used judiciously.

All RIG products are obtained from vaccinated humans or animals. There are at least 16 other lyssavirus species (also referred to as rabies-related viruses) and challenge studies have indicated that RABV-based vaccines do not confer protection against challenge with diverse lyssaviruses. It is generally accepted that RABV-based vaccines confer protection against phylogroup I lyssaviruses; however, challenge studies in animal models, have demonstrated varied levels of protection against different lyssavirus species which may be related to the vaccine strain used [21,22,23,24,25,26,27,28]. For example, reduced protection against Aravan virus (ARAV), Khujand virus (KHUV), and Irkut virus (IRKV) was noted for pre-vaccination and post-exposure prophylaxis [23]. For the European bat lyssaviruses and some phylogroup II viruses, the vaccine strain has been demonstrated to influence the level of protection [22,29]. Current rabies biologicals have been demonstrated to offer limited or no protection against disease with non-phylogroup I lyssaviruses [22,23,30,31,32]; however, cross-reaction may be possible with very high antibody titers [23]. From this, two important questions emerge. Can RIG neutralize diverse lyssaviruses? If yes, at what dose can RIG neutralize diverse lyssaviruses?

To address these questions, we investigated the ability of commercially available RIG to neutralize diverse lyssaviruses and assessed whether there is a dose-dependent effect.

## 2. Materials and Methods

### 2.1. Cells

Mouse neuroblastoma cells (MNA, C1300 clone, ECACC) were propagated in Dulbecco’s Modified Eagle Medium with Ham’s F12 (DMEM/F12, 1:1 mix, Biowest, Nuaillé, France) supplemented with 10% fetal bovine serum (Gibco, New York, NY, USA) and 1% antibiotics (100 U/mL penicillin, 100 µg/mL streptomycin and 0.25 µg/mL amphotericin B) (Lonza, Walkersville, MD, USA) and were incubated at 37 °C in an atmosphere containing 5% CO_2_.

### 2.2. Viruses

Where possible, a virus representative was selected for each lyssavirus species. At the time of this study, six lyssaviruses were not available, i.e., Bokeloh bat lyssavirus (BBLV), Gannoruwa bat lyssavirus (GBLV), KBLV, Lleida bat lyssavirus (LLEBV), Taiwan bat lyssavirus (TWBLV) and TWBLV-2. Cell culture supernatants of Australian bat lyssavirus (ABLV), European bat lyssavirus 1 (EBLV-1), European bat lyssavirus 2 (EBLV-2), and Ikoma lyssavirus (IKOV) were kindly provided by the Animal and Plant Health Agency, United Kingdom. Cell culture supernatants of ARAV, IRKV, KHUV, Lagos bat virus (LBV) lineage D, Shimoni bat virus (SHIBV), West Caucasian bat virus (WCBV) and RABV (*Desmodus rotundus-*, *Eptesicus fuscus-* and *Tadarida brasiliensis* strains) were kindly provided by the Centers for Disease Control and Prevention, United States of America. Brain material for RABV (canid and mongoose variants) were kindly provided by Agricultural Research Council-Onderstepoort Veterinary Research, South Africa. All other lyssaviruses (Table 1) were available at the Centre for Viral Zoonoses, University of Pretoria. All lyssaviruses were propagated and titrated in a Biosafety level 3 facility using MNA cells as previously described [33]. The identity of each isolate was confirmed by amplification and sequencing of the partial N-gene using the 001 lys and 550 B primers [34] as previously described [35].

### 2.3. Virus Titration

The virus titer was determined in duplicate in MNA cells using 8-well glass cell culture slides as previously described [49]. After incubation, the slides were fixed in cold acetone (Merck, Johannesburg, South Africa) for 30 min followed by staining with 7 µL of polyclonal fluorescein isothiocyanate anti-lyssavirus conjugate (N4–18, Rabies Unit, Agricultural Research Council-Onderstepoort Veterinary Research, Pretoria, South Africa) diluted 1:20 in phosphate-buffered saline (PBS, Lonza, USA) with Evans Blue (0.5% in PBS) as the counterstain. Slides were incubated at 37 °C in a humidified chamber for 30 min followed by two wash steps of 10 min in PBS. Slides were viewed under a fluorescent microscope under 200× magnification to determine the focus-forming dose (FFD_50_) [50].

### 2.4. Molecular Characterization

Once sufficient virus stocks were propagated, the sequences for the partial N-gene were determined to confirm viral identity. The complete glycoprotein sequence was also determined for the virus stocks used (refer to Table 1 for passage number) for the microneutralization test. Briefly, 140 µL cell culture supernatant was transferred to 560 µL AVL extraction buffer (Qiagen, Hilden, Germany) and 560 µL absolute ethanol for inactivation of the material. The manipulation and inactivation of infectious materials were performed in a biosafety 3 laboratory. The inactivated samples were removed from the containment laboratory for further manipulation under biosafety level 2 conditions and nucleic acids were extracted using the Viral RNA mini kit (Qiagen, Germany) according to the manufacturer’s instructions. Reverse transcription was performed on all samples using the following protocol. A volume of 5 µL RNA was added to 100 ng random hexamers (Integrated DNA Technologies, Coralville, IA, USA) and 1 µL dNTP mix (10 mM, Thermo Fisher Scientific, Vilnius, Lithuania) in a final volume of 12 µL.

This mixture was incubated at 65 °C for 5 min, followed by one-minute incubation on ice. Seven µL reaction mix containing 1× SSIV buffer (Invitrogen, Carlsbad, CA, USA), 5 M dithiothreitol (Invitrogen, USA), 40 U Ribolock RNase inhibitor (Thermo Fisher Scientific, Lithuania) and 200 U SuperScript IV reverse transcriptase (Invitrogen, USA) was added and incubated at 23 °C for 10 min, 50 °C for 30 min followed by inactivation at 80 °C for 10 min. All reactions were stored at −70 °C until use.

The partial N-gene was amplified as described previously [34,35,36,37,38,39,40,41,42,43,44,45,46] using the 001 lys and 550 B primers (Table 2). A single primer set was designed to amplify the complete G-gene of all lyssaviruses and internal primers were designed to allow determination of the complete G-gene using Sanger sequencing (Table 2). A volume of 5 µL cDNA was used in a 50 µL PCR reaction containing 0.4 µM of each primer, 0.2 mM dNTP mix (Thermo Fisher Scientific, Lithuania), 1.25 U DreamTaq DNA polymerase (Thermo Fisher Scientific, Lithuania), 1× DreamTaq Buffer (Thermo Fisher Scientific, Lithuania) and 1 mM MgCl_2_. The reactions were incubated at 94 °C for 1 min followed by 40 cycles of 94 °C for 30 s, 48 °C for 30 s, 72 °C for 3 min with a final extension step of 72 °C for 15 min. Products were analyzed on 1.5% (*w*/*v*) agarose gels and amplicons purified using the Wizard SV Gel and PCR Clean-Up system (Promega, Madison, WI, USA) according to the manufacturer’s instructions. Amplicons were sequenced using the Big Dye terminator v3.1 cycle sequencing kit (Thermo Fisher Scientific, Austin, TX, USA). Sequences were analyzed and concatenated using BioEdit sequence alignment editor version 7 [51].

### 2.5. Immunoglobulin

Two commercial preparations of immunoglobulin routinely used for rabies post-exposure management in South Africa were evaluated; human rabies immunoglobulin (Rabigam, National Bioproducts Institute, Pinetown, South Africa) supplied at a concentration of 150 IU and equine rabies immunoglobulin (Equirab, Bharat serums and vaccines limited, Ambernath, India) supplied at a concentration of 300 IU/mL. The WHO Second International Rabies Immunoglobulin Reference Standard, diluted to a potency of 2 IU/mL, was also included [52].

### 2.6. Microneutralization Test

To assess the ability of commercial immunoglobulins to neutralize lyssaviruses, a microneutralization test, based on the rapid fluorescent focus inhibition test, was used [49,53]. Immunoglobulin was tested at two concentrations, the concentration as supplied by the manufacturer, i.e., Rabigam at 150 IU/mL and Equirab at 300 IU/mL, and both preparations were diluted in PBS to a potency of 2 IU/mL. In brief, 1.75 µL of immunoglobulin was mixed in the first well of an 8-well glass cell culture slide (Marienfield, Lauda-Königshofen, Germany) with 7 µL of DMEM/F12 media. From this initial dilution, 1.75 µL was transferred to another well and mixed with 7 of DMEM/F12 media, which was repeated until a 1:781250 dilution was reached. To each well, 7 µL virus (at 50 FFD_50_ as determined by titration) was added and incubated in a humidity chamber at 37 °C for 90 min. Thereafter, 14 MNA cells (at 2 × 10^6^ cells/mL) were added to each well and incubated in a humidity chamber at 37 °C for 24 h. After incubation, slides were fixed and stained as described for virus titration. For each well, 10 fields were observed and the 50% endpoint neutralizing titers were calculated by the Reed and Muench method [50].

### 2.7. Antigenic Cartography

A lyssavirus antigenic map was generated from the microneutralization test data as previously described [54,55] using the following online software available at https://acmacs-web.antigenic-cartography.org/ (accessed on 17 January 2023). End-point titers for each virus for each immunoglobulin tested (including different concentrations) were included and an antigenic map was constructed in three dimensions with multiple random restart optimizations (*n* = 100).

### 2.8. Comparison of Antigenic and Genetic Data

Glycoprotein gene sequences generated in this study (Section 2.4) were used together with publicly available sequencing data for vaccine strains, i.e., Pitman-Moore strain (GenBank accession number: AJ871962) and Flury low-egg passage strain (GenBank accession number: GU565703) to create a multiple alignment using BioEdit sequence alignment editor version 7 [51]. Phylogenetic analysis and evolutionary distance calculations were performed using Molecular Evolutionary Genetic Analysis software version 11.0.11 (MEGA11) [56]. Pairwise comparisons of data were performed using the Pearson product–moment correlation coefficient in Microsoft Excel.

## 3. Results

### 3.1. Characterization of Antigenic Sites

For each of the isolates used, the complete G-gene sequence (Supplementary File S1) and amino acid conservation for each of the antigenic sites [57,58] were determined. Based on amino acid conservation of the G-gene ectodomain, the fixed CVS-11 strain of RABV had the lowest number of substitutions (*n* = 5) compared to the vaccine strains. The most divergent virus in phylogroup I was IRKV with 105 substitutions. In phylogroup II, LBV lineage A was the most divergent, with 166 amino acid substitutions, and IKOV was the most divergent for the unassigned viruses, with 203 substitutions, compared to the vaccine strains (Table S1). The antigenic domain conservation ranged from 61.8–97.1% for phylogroup I, 47.1–56% for phylogroup II and was only conserved at 35.3% of sites for the unassigned viruses (Table 3).

### 3.2. Neutralization Activity

**Figure 1:**
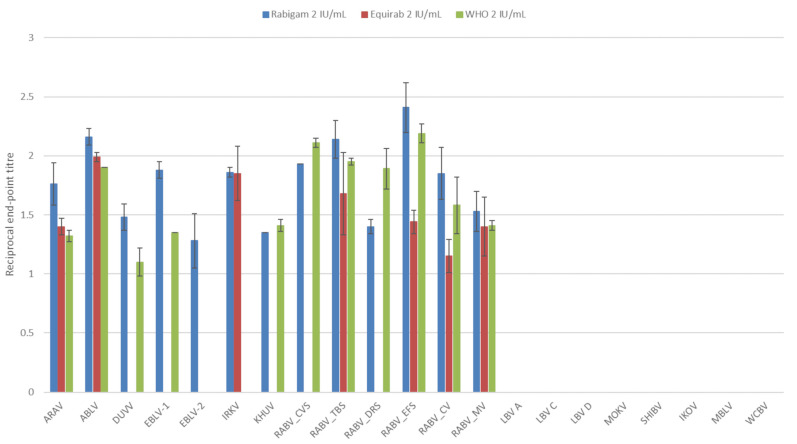
The average log-transformed reciprocal neutralization titers of the reference standard, hRIG and eRIG at 2 IU/mL, tested against lyssaviruses in triplicate using a microneutralization test. Error bars indicate standard deviation. Virus abbreviations: Aravan virus (ARAV), Australian bat lyssavirus (ABLV), Duvenhage virus (DUVV), European bat lyssavirus 1 (EBLV-1), European bat lyssavirus 2 (EBLV-2), Irkut virus (IRKV), Khujand virus (KHUV), rabies virus Challenge virus standard (RABV_CVS-11), rabies virus *Tadarida brasiliensis* strain (RABV_TBS), rabies virus *Desmodus rotundus* strain (RABV_DRS), rabies virus *Eptesicus fuscus* strain (RABV_EFS), rabies virus canid variant (RABV_CV), rabies virus mongoose variant (RABV_MV), Lagos bat virus lineage A (LBV A), Lagos bat virus lineage C (LBV C), Lagos bat virus lineage D (LBV D), Mokola virus (MOKV), Shimoni bat virus (SHIBV), Ikoma lyssavirus (IKOV), Matlo bat lyssavirus (MBLV) and West Caucasian bat virus (WCBV).

At a low concentration (2 IU/mL), only phylogroup I lyssaviruses were neutralized. Overall, the hRIG displayed the highest neutralization activity with all phylogroup I viruses neutralized (Figure 1). As expected, the highest neutralization titers were observed for the RABV strains and ABLV. Not all phylogroup I viruses were neutralized by the WHO reference standard or equine rabies immunoglobulin (eRIG). The WHO reference standard was unable to neutralize EBLV-2 and IRKV. The eRIG was unable to neutralize DUVV, EBLV-1, EBLV-2, KHUV, RABV (CVS-11), and RABV (*Desmodus rotundus* strain). Statistically significant (*p* < 0.05) differences between the average neutralization titers determined for eRIG and hRIG were observed.

**Figure 2:**
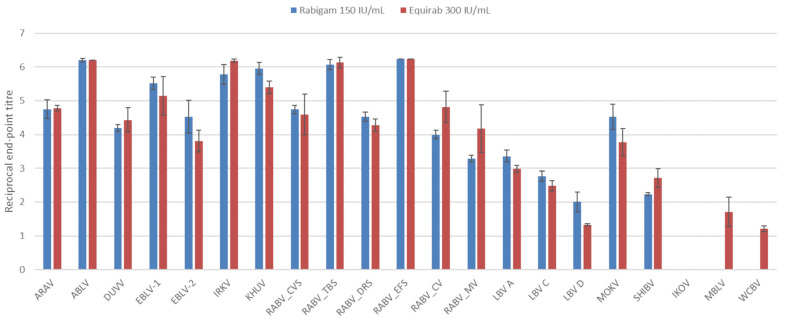
The average log-transformed reciprocal neutralization titers of hRIG and eRIG, at 150 IU/mL and 300 IU/mL, respectively, tested against lyssaviruses in triplicate using a microneutralization test. Error bars indicate standard deviation. Virus abbreviations: Aravan virus (ARAV), Australian bat lyssavirus (ABLV), Duvenhage virus (DUVV), European bat lyssavirus 1 (EBLV-1), European bat lyssavirus 2 (EBLV-2), Irkut virus (IRKV), Khujand virus (KHUV), rabies virus Challenge virus standard (RABV_CVS-11), rabies virus *Tadarida brasiliensis* strain (RABV_TBS), rabies virus *Desmodus rotundus* strain (RABV_DRS), rabies virus *Eptesicus fuscus* strain (RABV_EFS), rabies virus canid variant (RABV_CV), rabies virus mongoose variant (RABV_MV), Lagos bat virus lineage A (LBV A), Lagos bat virus lineage C (LBV C), Lagos bat virus lineage D (LBV D), Mokola virus (MOKV), Shimoni bat virus (SHIBV), Ikoma lyssavirus (IKOV), Matlo bat lyssavirus (MBLV) and West Caucasian bat virus (WCBV).

At higher concentrations, neutralization was observed for all viruses except IKOV (Figure 2). Although the concentration of eRIG was double that of hRIG, the difference in neutralizing titers was not significant (*p* > 0.05). Neutralizing titers for phylogroup I viruses were similar for both immunoglobulin preparations; however, neutralizing titers for non-phylogroup I lyssaviruses were slightly higher for eRIG compared to hRIG. The highest neutralizing titers were obtained for the RABV *Eptesicus fuscus* strain in phylogroup I, Mokola virus (MOKV) in phylogroup II, and MBLV for the unassigned viruses. Multiple virus isolates for RABV and LBV were included and variations in the neutralization titers were observed. The highest neutralization titers for both hRIG and eRIG were observed for the RABV *Eptesicus fuscus* strain and the lowest with the RABV mongoose variant. For the LBV isolates, the highest neutralizing titer was observed for lineage A followed by lineage C with the lowest titer for lineage D for both immunoglobulin preparations.

### 3.3. Antigenic Cartography

**Figure 3:**
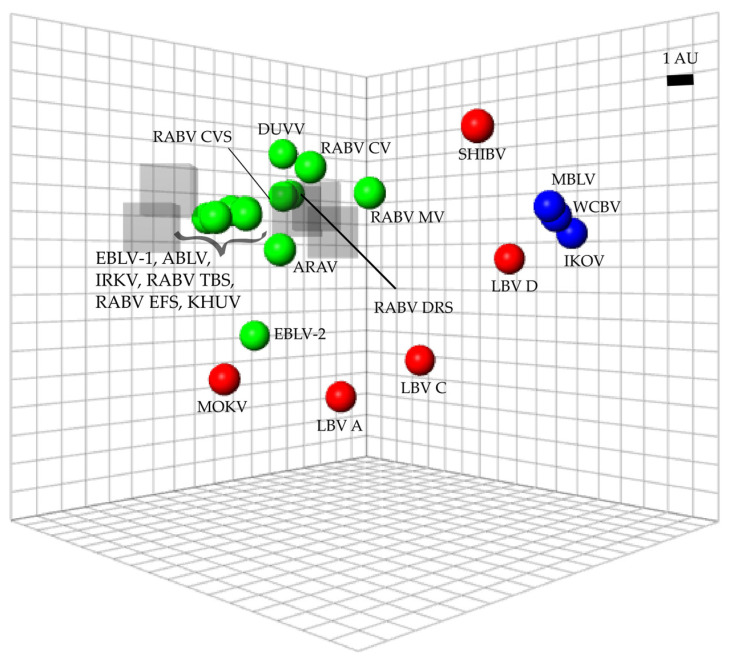
Antigenic map of lyssaviruses and RIG. The distance from each serum to each virus was determined using the neutralization titer (data for all immunoglobulins at all concentrations were included). Multidimensional scaling allows the positioning of sera and viruses relative to each other with free orientation within the map axes. The scale bar indicates one antigenic unit (AU), equivalent to a two-fold dilution in antibody titer. Immunoglobulins are indicated by translucent grey boxes, phylogroup I lyssaviruses are indicated in green circles, phylogroup II lyssaviruses are indicated in red circles and unassigned lyssaviruses are indicated in blue circles. Virus abbreviations: Aravan virus (ARAV), Australian bat lyssavirus (ABLV), Duvenhage virus (DUVV), European bat lyssavirus 1 (EBLV-1), European bat lyssavirus 2 (EBLV-2), Irkut virus (IRKV), Khujand virus (KHUV), rabies virus Challenge virus standard (RABV_CVS-11), rabies virus *Tadarida brasiliensis* strain (RABV_TBS), rabies virus *Desmodus rotundus* strain (RABV_DRS), rabies virus *Eptesicus fuscus* strain (RABV_EFS), rabies virus canid variant (RABV_CV), rabies virus mongoose variant (RABV_MV), Lagos bat virus lineage A (LBV A), Lagos bat virus lineage C (LBV C), Lagos bat virus lineage D (LBV D), Mokola virus (MOKV), Shimoni bat virus (SHIBV), Ikoma lyssavirus (IKOV), Matlo bat lyssavirus (MBLV) and West Caucasian bat virus (WCBV).

Each lyssavirus and RIG preparation were assigned a position on an antigenic map such that the distance between the virus and the RIG directly corresponded to the neutralization titer from the microneutralization test (Figure 3). As a result, the higher the neutralization titer, the shorter the antigenic distance. Antigenic distance was indicated in antigenic units (AU) with 1 AU equal to a two-fold change in titer. Lyssaviruses of phylogroup I were antigenically closely related with an average distance of 3.8 AU (Table S2). Within phylogroup I, ABLV and RABV (*Tadarida brasiliensis* strain) were antigenically the most similar (0.11 AU) while EBLV-2 and RABV (mongoose variant) were antigenically the most divergent (7.94 AU). Within phylogroup II, LBV lineage A and LBV lineage C were antigenically the most similar (3.55 AU), while MOKV and SHIBV were antigenically the most diverse (13.72 AU). Within the unassigned viruses, WCBV and MBLV were antigenically the most similar (0.61 AU), while IKOV and WCBV were antigenically the most diverse (1.17 AU). Intra-phylogroup antigenic distances ranged from 2 to 14.9 AU. Antigenic distances between phylogroup I and RIG were the lowest for RABV CVS-11, while EBLV-2 and RABV (mongoose variant) were antigenically the most diverse. For phylogroup II, MOKV was antigenically the most similar to RIG, while LBV lineage D was antigenically the most diverse. For the unassigned viruses, MBLV was antigenically the most similar to RIG, while IKOV was antigenically the most diverse. On average, the antigenic distance between RIG and phylogroup I was 4.02 AU, 8.94 AU for phylogroup II, and 12.03 AU for the unassigned viruses.

### 3.4. Comparison of Genetic and Antigenic Data

**Figure 4:**
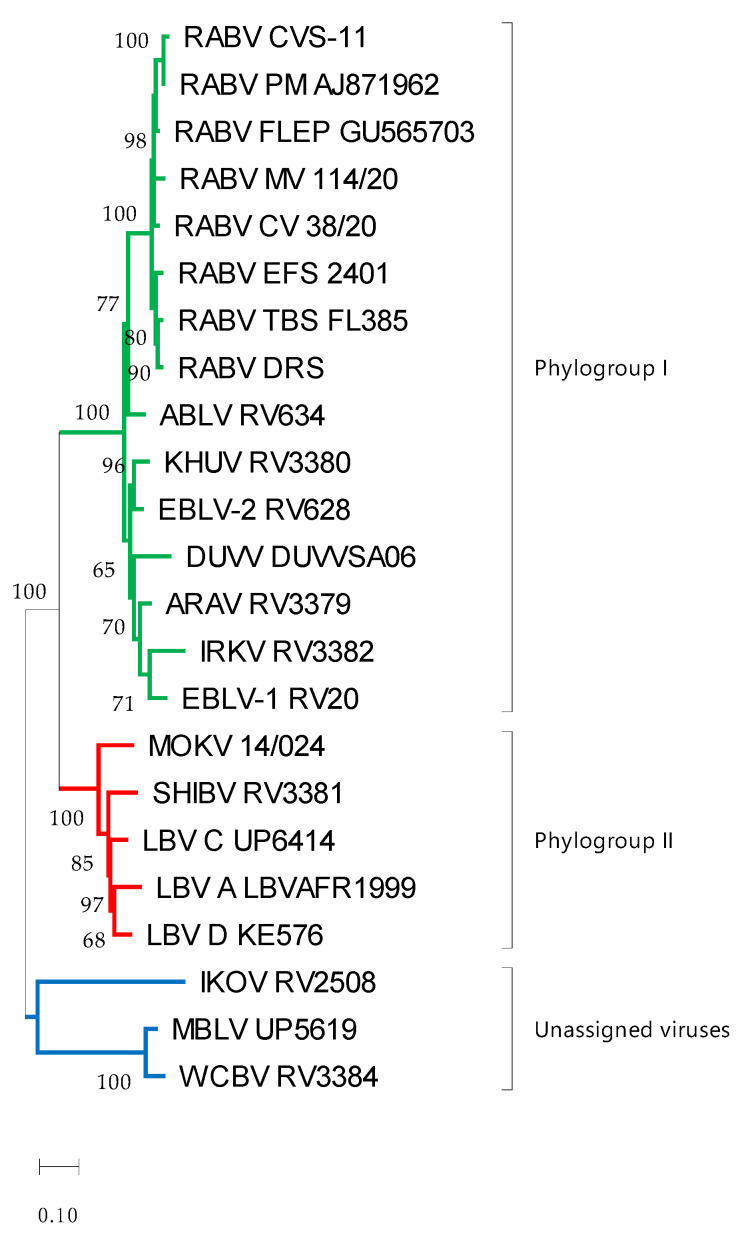
Maximum likelihood phylogenetic tree of the ectodomain of lyssaviruses using the general time-reversal model with invariant sites and a gamma distribution of rates across sites. The reliability of the branching pattern was evaluated with 1000 replications and is indicated at nodes if above 50. The scale bar indicates substitutions per site. Phylogroup I is indicated in green, phylogroup II is indicated in red and the unassigned viruses are indicated in blue. Virus abbreviations: Aravan virus (ARAV), Australian bat lyssavirus (ABLV), Duvenhage virus (DUVV), European bat lyssavirus 1 (EBLV-1), European bat lyssavirus 2 (EBLV-2), Irkut virus (IRKV), Khujand virus (KHUV), rabies virus Challenge virus standard (RABV_CVS-11), rabies virus *Tadarida brasiliensis* strain (RABV_TBS), rabies virus *Desmodus rotundus* strain (RABV_DRS), rabies virus *Eptesicus fuscus* strain (RABV_EFS), rabies virus canid variant (RABV_CV), rabies virus mongoose variant (RABV_MV), Lagos bat virus lineage A (LBV A), Lagos bat virus lineage C (LBV C), Lagos bat virus lineage D (LBV D), Mokola virus (MOKV), Shimoni bat virus (SHIBV), Ikoma lyssavirus (IKOV), Matlo bat lyssavirus (MBLV) and West Caucasian bat virus (WCBV).

Differences between antigenic and phylogenetic relationships were observed. For example, phylogenetically, the mongoose variant of RABV was closely related to CVS-11 and the RABV vaccine strains (Figure 4); however, antigenically it was one of the viruses most distant to CVS-11, along with EBLV-2. Similarly, LBV lineage A and LBV lineage D appeared to be phylogenetically closely related, but antigenically LBV lineage A was closer to MOKV than LBV lineage D.

**Figure 5:**
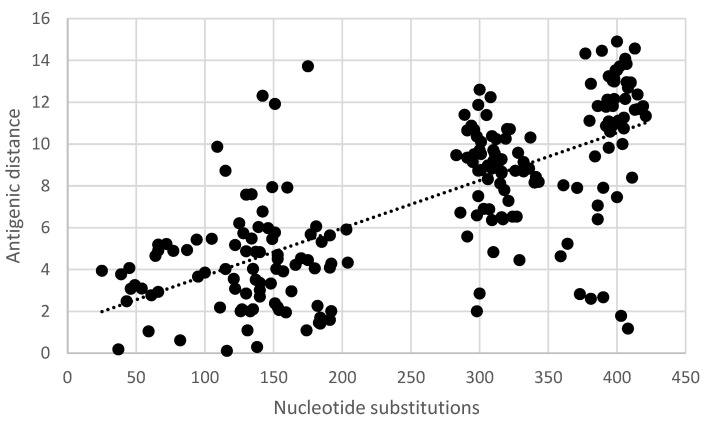
Pair-wise correlation of antigenic and genetic distance. Antigenic distances are indicated in antigenic units and genetic distances are indicated as the number of nucleotide substitutions in the ectodomain of the G-gene.

**Figure 6:**
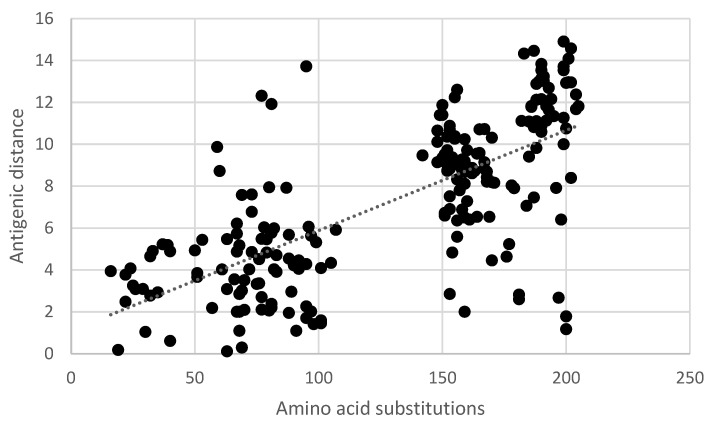
Pair-wise correlation of antigenic and genetic distance. Antigenic distances are indicated in antigenic units and genetic distances are indicated as the number of amino acid substitutions in the ectodomain of the G-gene.

**Figure 7:**
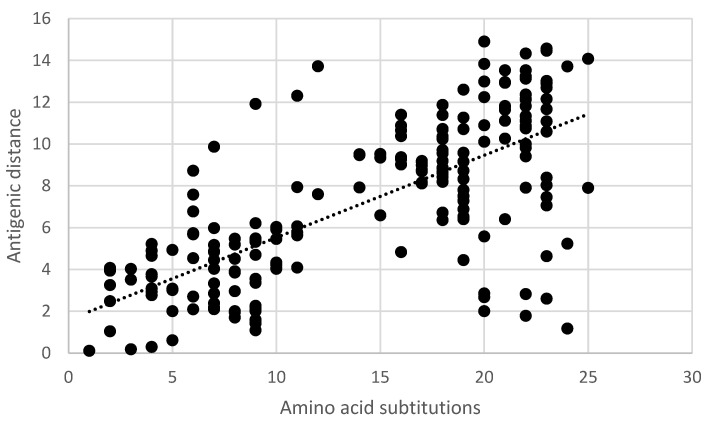
Pair-wise correlation of antigenic and genetic distances. Antigenic distances are indicated in antigenic units and genetic distances are indicated as the number of amino acid substitutions in known antigenic sites.

Pair-wise comparisons of the antigenic distance and the nucleotide (Table S3) and amino acid substitutions (Table S1) of the ectodomain of the G-gene indicated a statistically significant correlation (Pearson product-moment correlation coefficient r = 0.7, 95% confidence interval 0.63–0.77, *p* < 0.05) (Figure 5 and Figure 6). Similarly, a statistically significant correlation (Pearson product-moment correlation coefficient r = 0.7, 95% confidence interval 0.63–0.77, *p* < 0.05) was observed for a pair-wise comparison of the antigenic distance and the amino acid substitutions of known antigenic sites (Table S4, Figure 7).

## 4. Discussion

Rabies remains a neglected disease mostly affecting developing countries and conservatively causes more than 59,000 human deaths every year [5]. Rabies virus infection and subsequent disease in humans can be prevented through the prompt application of rabies PEP. The management of cases with strict adherence to WHO rabies PEP guidelines [6] results in close to 100% efficacy [59]. The use of RIG is recommended in all category III exposures i.e., single or multiple transdermal bites or scratches, contamination of mucous membranes or broken skin with saliva from animal licks, and exposures due to direct contact with bats [6]. Rabies vaccines and RIG have been available for decades; however, these biologicals are based on RABV only. The known diversity of lyssavirus species has more than doubled over the years with a growing concern for the efficacy of the currently available biologicals against the range of viruses that are antigenically distinct from RABV. Existing investigations have suggested that the available biologicals will not be effective against all lyssaviruses [23,60,61,62]. Thus, this study aimed to investigate the cross-lyssavirus neutralization of two RIG formulations available in South Africa.

A limitation of all in vitro studies utilizing virus isolates propagated in cell culture is the potential genetic shift or drift driven by the culture conditions. To address this limitation, for each isolate used in this study, the complete G-gene sequence was determined after passage to ensure a valid comparison of antigenic and genetic data. This, however, does not rule out any potential differences between the original viruses and the cell culture-propagated challenge viruses, although the G-gene is considered genetically stable even after several passages [63]. The G of lyssaviruses forms a homo-trimer on the surface of the virion and plays a crucial role in the attachment to host cell receptors and induces an immune response [58]. The sites that are important for the neutralization of RABV have previously been determined using monoclonal antibodies [57,58]. Amino acid substitutions were observed for most of the antigenic sites across the phylogroups and the unassigned lyssaviruses. Only site IV (amino acid 251) was conserved across phylogroups I and II with different amino acids present for each of the unassigned viruses. Site G1 (amino acids 342–343) was also conserved across phylogroups I and II as well as two of the unassigned viruses, while IKOV was the only lyssavirus not conserved at this site. Although these sites are important in RABV neutralization, there is limited data to suggest that the same sites have a similar function in the neutralization of other lyssaviruses. In the case of polyclonal sera, it may be that uncharacterized epitopes could influence the repertoire of neutralizing antibodies following vaccination.

Commercial RIG, when used at low concentrations, was able to exclusively neutralize viruses that belong to phylogroup I. Unexpectedly, at a concentration of 2 IU, the eRIG preparation tested could not neutralize all phylogroup I lyssaviruses. This result requires a more comprehensive evaluation and should include the testing of different RIG batches from different manufacturers. A range of lower concentrations should be included with verification of the potency before testing. Various factors may affect the ability of antibodies to neutralize lyssaviruses, which include but may not be limited to the mechanism of neutralization, steric hindrances for epitopes that are in close proximity, competition for binding to antigenic sites, changes in the antigenic sites and the immunodominant antigenic sites that are important for the neutralization of different lyssaviruses [58,64,65,66]. In addition, a critical number of immunoglobulins are required to allow lyssavirus neutralization, with less than 270 IgG or 40 IgM molecules per virion in polyclonal sera being unable to mediate the neutralization of CVS [66]. Since there is no WHO prequalification for polyclonal sera preparations such as RIG, batch-to-batch variations can occur, raising concerns regarding the quality of some of these preparations [67]. Historically, potency testing was performed using the mouse neutralization test; however, this test is no longer recommended due to ethical considerations [33]. Alternative tests include virus neutralization assays, such as the RFFIT, fluorescent antibody virus neutralization test, or validated enzyme-linked immunosorbent assays (ELISA) [33,34,35,36,37,38,39,40,41,42,43,44,45,46,47,48,49,50,51,52,53,54,55,56,57,58,59,60,61,62,63,64,65,66,67,68]. Virus neutralization tests quantify the virus neutralizing antibodies against CVS, while ELISAs either detect all binding antibodies or only virus neutralizing antibodies against vaccine strains depending on their design [69,70]. Although there is a strong correlation between the antibody titers determined using virus neutralization tests and ELISAs, the antibody titers obtained using ELISA are typically higher and more variable compared to RFFIT [69,70]. A study on the potency of veterinary vaccines in Sri Lanka demonstrated that the potency of some vaccines is lower than stated by the manufacturer [71]. The potency of some RIG preparations may therefore be lower than stated by the manufacturer, especially if ELISA was used with a vaccine strain as the capture antigen. In addition, the potency can be impacted by the short shelf-life of the product, especially if the cold chain was not properly maintained. As a result, inadequate neutralization could occur at low concentrations; however, at higher concentrations the above-mentioned factors appeared to be overcome and cross-neutralization was observed across all phylogroups. This dose-dependent cross-neutralization has been reported in previous studies [23,60,61]. At higher doses, hRIG could neutralize phylogroup II viruses in addition to phylogroup I viruses, but no neutralization was observed for the unclassified viruses. A similar trend was observed for the eRIG, which could also neutralize some of the unclassified viruses, i.e., WCBV and MBLV. Both commercial RIGs were tested at the concentration supplied by the manufacturer and eRIG was therefore used at double the concentration of the hRIG, similar to PEP recommendations, to accommodate the decreased half-life of eRIG compared to hRIG. The neutralization observed could therefore be due to a dose-dependent effect or could be due to inherent differences between RIG produced in humans and equines, such as different virus strains used in vaccines or individual variation in immune reactivity. For example, horses have the highest number of IgG constant region genes described for mammals to date [72] and since the constant region is thought to play a central role in the interactions of antibodies and antigens [73], suggests that horses produce a larger diversity of antibodies that may bind more diverse lyssaviruses.

The antigenic differences between viruses will determine to which degree immunity is induced. Antigenic cartography is a computational method that facilitates reliable quantitative interpretation and visualization of neutralization data [55] with a previously determined average prediction error of 1.22 AU in three dimensions [54]. The antigenic map generated in this study was congruent with previously published data [27,28,29,30,31,32,33,34,35,36,37,38,39,40,41,42,43,44,45,46,47,48,49,50,51,52,53,54]. Phylogroup I lyssaviruses are antigenically similar; however, CVS-11 was distinct from other wild-type RABV strains except for the *Desmodus rotundus* strain. Differences between antigenic and phylogenetic relationships were also apparent, emphasizing that antigenic relationships cannot be predicted by sequence data alone [54]. Based on the antigenic distances between the viruses evaluated and RIG, a 16-fold higher titer would be required to neutralize all phylogroup I viruses, a 360-fold higher titer to neutralize all phylogroup II viruses and a 4182-fold higher titer to neutralize all unassigned viruses. A limitation of this work is that not all known lyssaviruses were available at the time of this study. However, based on previous antigenic maps, it is likely that RIG will neutralize BBLV, GBLV, TWBLV, and KBLV at similar levels as observed for other phylogroup I viruses [27], with limited neutralization of LBV lineage B similar to MOKV [54] and with little to no neutralization of LLEBV [74].

Statistically significant correlations between evolutionary (nucleotide and amino acid substitution) and antigenic distances were observed. However, similar to a previous report, approximately 30% of the variation observed cannot be explained by genetic data [54].

Rabies is fatal once patients become symptomatic, and the prevention of disease should, therefore, be the focal point when treating exposed patients. Numerous studies [16,17,18,31] have indicated that vaccination alone, especially in cases with severe exposure, is not always reliable, and RIG should therefore always be administered when indicated. However, RIG is prohibitively expensive and in constant short supply. For example, in South Africa, rabies is a notifiable medical condition and rabies PEP is provided at no cost to the patient at public health institutions. These cases are managed according to a risk-based approach due to supply shortages of RIG [75]. Currently, the direct PEP cost is approximately $198 (USD) for four doses of the rabies vaccine and one vial of RIG. This places a significant financial burden on healthcare facilities to procure the needed biologicals, for example, over a 3-year period, the KwaZulu-Natal province in South Africa reported >130,000 human rabies PEP cases [76]. A review of human rabies cases in South Africa over a 10-year period (2008–2018) indicated that 39% of individuals seeking medical treatment after exposure did not receive RIG, although indicated [77]. The limited availability and costs involved (direct and indirect) of rabies PEP are major contributing factors to ineffective post-exposure management of patients in the majority of developing countries [5,78,79,80,81,82,83,84]. Complicating the matter further is the description of novel and genetically diverse lyssaviruses. The commercial RIG preparations evaluated in this study were able to neutralize phylogroup I lyssaviruses, even at low concentrations. However, the neutralization of more diverse lyssaviruses outside phylogroup I was dose-dependent, and, in most cases, the neutralization titers were far below those observed for phylogroup I, even when applied at higher doses. This implies that following the current guidelines for the calculation of the RIG dose to be administered based on body weight alone may result in some patients receiving an insufficient dose of RIG to allow the neutralization of diverse lyssaviruses. Only a few cases of rabies in humans have reportedly been caused by rabies-related viruses; however, in many African countries, reporting human rabies cases is not mandatory [79] with up to 95% of human cases going unreported in eastern and southern Africa [81]. Another critical issue contributing to limited epidemiological data in resource-limited settings is the lack of capability and capacity to diagnose rabies and to routinely characterize rabies-positive samples [5,46,79,81]. Several spillover infections caused by rabies-related viruses have been reported in domestic animals, which increases the risk of human exposure [44,46,85,86]. Thus, the lack of protection against some of these viruses using the current PEP guidelines is concerning, especially in developing countries where multiple lyssaviruses are known to circulate. Taken together, difficulties in the supply of RIG, inhibitory costs, and the sub-optimal efficacy against diverse lyssaviruses, suggest that the time has come to intensify efforts to establish alternatives such as monoclonal antibody cocktails. Substituting RIG for monoclonal antibodies in rabies PEP management can result in lower costs, improved availability, increased stability, safety, and efficacy and may in the future also have an application in the therapeutic treatment of rabies in humans [84,87].

## Figures and Tables

**Table 1:** Details of lyssaviruses propagated for evaluation of commercial rabies immunoglobulin.

Virus	Reference Number	Host	Year	Country	Passage Number	GenBank Accession Number	References
Australian bat lyssavirus	RV634	Fruit bat	1996	Australia	7	AY062067	[36]
Aravan virus	RV3379	*Myotis blythi*	1991	Kyrgyzstan	12	EF614259	[37]
Duvenhage virus	DUVVSA06 SPU94.06	*Homo sapiens*	2006	South Africa	Unknown	EU623444	[38]
European bat lyssavirus 1	RV20	*Eptesicus serotinus*	1986	Denmark	11	KF155003	[39]
European bat lyssavirus 2	RV628 18/96	*Myotis daubentonii*	1996	United Kingdom	8	KY688136	[40]
Ikoma lyssavirus	RV2508	*Civettictis civetta*	2009	Tanzania	8	NC018629	[41]
Irkut virus	RV3382	*Murina leucogaster*	2002	Russia	5	NC020809	[42]
Khujand virus	RV3380	*Myotis mystacinus*	2001	Tajikistan	8	NC025385	[37]
Lagos bat virus (Lineage A)	LBVAfr1999	*Rousettus* sp.	1999	France (imported)	Unknown	EF547447	[43]
Lagos bat virus (Lineage C)	UP6414	*Epomophorus wahlbergi*	2016	South Africa	17	MH643893	[44]
Lagos bat virus (Lineage D)	KE576	*Rousettus aegyptiacus*		Kenya	4	GU170202	[45]
Mokola virus	14/024	*Felis catus*	2014	South Africa	12	KP899612	[46]
Matlo bat lyssavirus	UP5619	*Miniopterus natalensis*	2015	South Africa	15	MW653808	[2]
Rabies virus	CVS-11	Cattle	1882	France	Unknown	GQ918139	
Rabies virus (*Desmodus rotundus* strain)	Not available	*Desmodus rotundus*	Unknown	Brazil	7	AF070449	[47]
Rabies virus (*Eptesicus fuscus* strain)	2401	*Urocyon cinereoargenteus*	2009	United States of America	12	JQ685934	[48]
Rabies virus (*Tadarida brasiliensis* strain)	FL385	*Tadarida brasiliensis*	2003	United States of America	8	JQ685905	[48]
Rabies virus (canid variant)	38/20	*Canis familiaris*	2020	South Africa	11	OR067379	This study
Rabies virus (mongoose variant)	114/20	Mongoose	2020	South Africa	12	OR067380	This study
Shimoni bat virus	RV3381	*Macronycteris vittatus*	2009	Kenya	11	NC025365	[45]
West Caucasian bat virus	RV3384	*Miniopterus shreibersii*	2002	Russia	7	NC025377	[42]

**Table 2:** Primers used for the amplification of the partial nucleoprotein gene and complete glycoprotein gene of lyssaviruses.

Primer	Sequence (5′-3′) ^1^	Use	Position ^2^
001 lys	ACGCTTAACGAMAAA	Forward primer, partial N-gene PCR	1–15
550 B	GTRCTCCARTTAGCRCACAT	Reverse primer, partial N-gene PCR	647–666
GlycoF	TGGTGYATNAAYATRAAYC	Forward primer, G-gene PCR	3000–3018
GlycoR	GGRGARTTNARRTTRTARTC	Reverse primer, G-gene PCR	5520–5539
GF1	GAYCCNAGRTAYGARGARTC	Forward sequencing primer	3687–3706
GF2	ATNCCNGARATGCARTC	Forward sequencing primer	4491–4507
GF3	CWTCNTGGGARTYNTAYAA	Forward sequencing primer	4849–4867

^1^ M = A or C, R = A or G, Y = C or T, N = any base, W = A or T; ^2^ Numbered according to rabies virus, Pasteur virus strain, GenBank accession number: M13215.

**Table 3:** Amino acid conservation for antigenic sites for representative lyssaviruses analyzed in this study and vaccine strains.

Virus	Site IIb (34–42)	Site IIa (198–200)	Site I (226–231)	Site IV (251)	Site G5 (261–264)	Site III (330–338)	Site G1 (342–343)
Pitman-Moore vaccine strain	GCTNLSEFS	KRA	KLCGVL	W	HDFR	KSVRTWNEI	KG
Flury low egg passage vaccine strain	GCTNLSEFS	KRA	KLCGVL	W	HDGR	KSVRTWNEI	KG
Australian bat lyssavirus	GCTSLSGFS	KKA	KLCGIS	W	HDFH	KSVRTWNEI	KG
Aravan virus	GCTTLTAFS	KKA	KLCGVM	W	HDFH	KSVREWTEV	KG
Duvenhage virus	GCTTLTPFS	KKA	RLCGIS	W	HDFH	KSVREWKEI	KG
European bat lyssavirus 1	GCTTLTPFS	KKA	RLCGVP	W	HDFH	KSVREWKEV	KG
European bat lyssavirus 2	GCTTLTVFS	KKA	KLCGIS	W	HDFH	KSIREWTDV	KG
Ikoma lyssavirus	GCNEGSKVS	ILL	IICGKS	M	HTVK	KSVDNWTDI	PI
Irkut virus	GCTTLTAFN	KKA	KLCGMA	W	HDFH	KSIREWKEI	KG
Khujand virus	GCTTLSGFT	KRA	KLCGVS	W	HDFH	KSIREWSEI	KG
Lagos bat virus (Lineage A)	GCSETSSFT	RKA	TLCGKP	W	HNNR	KRVDNWVDI	KG
Lagos bat virus (Lineage C)	GCSDTATFS	KKS	TLCGKP	W	HNNR	LRVDSWNDI	KG
Lagos bat virus (Lineage D)	GCSTSTSFS	RKA	TLCGKP	W	HNNR	RRVDNWTDI	KG
Mokola virus	GCNTESPLT	RMA	TLCGKP	W	HNDR	KRVDRWADI	KG
Matlo bat lyssavirus	DCTSEQSIT	KLV	SICGRQ	A	HDIK	IKVENWSDI	KG
Rabies virus (Challenge virus standard)	GCTNLSEFS	KRA	KLCGVL	W	HDFH	KSVRTWNEI	KG
Rabies virus (*Desmodus rotundus* strain)	GCTNLSGFS	KKA	KLCGVL	W	HDFH	KSVRTWNEI	KG
Rabies virus (*Eptesicus fuscus* strain)	GCTSLSGFS	KRA	KLCGVP	W	HDFH	KSIRTWNEI	KG
Rabies virus (*Tadarida brasiliensis* strain)	GCTSLSGFS	KKA	KLCGVS	W	HDFH	KSVRTWNEI	KG
Rabies virus (canid variant)	GCTNLSGFS	KRA	KLCGVL	W	HDFR	KSVRTWNEI	KG
Rabies virus (mongoose variant)	GCTNLSGFS	KRA	KLCGVL	W	HDFR	KSIRTWDEI	KG
Shimoni bat virus	GCSSSSTFS	KKS	TLCGKP	W	HNNR	KRVDRWEEI	KG
West Caucasian bat virus	YCTTEQSIT	KLV	SICGRQ	V	HDIK	IKVENWSEV	KG

## Data Availability

The data presented in this study are contained in this article or Supplementary Materials.

## Supplementary Materials

The following supporting information can be downloaded at: https://www.mdpi.com/article/10.3390/vaccines11071255/s1, Supplementary File S1: Complete glycoprotein coding sequences for lyssaviruses used in this study; Table S1: Number of amino acid substitutions of the glycoprotein ectodomain between lyssaviruses; Table S2: Antigenic distances between lyssaviruses and RIG; Table S3: Number of nucleotide substitutions of the glycoprotein ectodomain between lyssaviruses; Table S4: Number of amino acid substitutions of known antigenic sites of lyssaviruses.
