# Supplementary material for: Comparative Neutralization Activity of Commercial Rabies Immunoglobulin against Diverse Lyssaviruses
Source: Vaccines (Basel). 2023 Jul 18;11(7):1255. doi: 10.3390/vaccines11071255 (PMC10383743; doi:10.3390/vaccines11071255)
Supplement: Supplementary file 1 [file vaccines-11-01255-s001.zip › Supplementary file 1 Complete glycoprotein seqeunces for lyssaviruses.pdf]

>ARAV_RV3379_Complete glycoprotein cds_P#12

ATGCCCCTTCAGGCAATTCCACTCTTCTCCCTCATTCTTCCCGTTTTAGTGGCAGGGAAGTTCCCCATATATACT
ATCCCTGACAAGATCGGTCCTTGGAGTCCCATCGACATCAACCACCTCAGATGCCCAAACAATCTGGTTGTAG
AAGACGAAGGATGTACAACTCTCACTGCGTTCTCTTATATGGAGTTGAAAGTGGGGTATATCACCACTATAAA
AGTGAGTGGGTTTACATGTACTGGGGTCGTTACAGAGGCTGAGACTTACACCAACTTCGTGGGATATGTTACA
ACCACCTTTCGGCGCAAGCATTTCAGGCCGACCGCAAGTGCCTGCAGAGAGGCGTACAACTGGAAAGCTACA
GGTGATCCCAGATACGAGGAGTCTCTGCATAACCCTTACCCCGACTCCCACTGGTTAAGAACAGTCAAGACCA
CAAAGGAATCACTCCTAATTATATCTCCGAGTGTGGCAGATATGGACGCATATGACAAAGCACTCTACTCTAA
GATATTCCCCAACGGAAAATGCTTAGGAGTGTCCTTGAGCTCCCCTTTCTGCTCGACCAATCACGATTACACCC
TTTGGATGCCTGAGAATCCGAAACCAGGTGTATCATGTGACATCTTCACCACAAGCAAGGGGAAGAAGGCAA
CGAAGGACGGCAAGCTGTGCGGGTTCGTCGACGAGAGAGGACTTTATAAATCATTGAAAGGGGCATGTAAA
CTGAAACTCTGCGGGGTCATGGGATTGAGGCTGATGGATGGGTCCTGGGTGTCTCTCCAAAAGACAGAGGAG
TCTGAATGGTGTTCTCCGAATCAACTGATTAACATTCATGACTTCCACTCAGATGAGATTGAACACATGGTGGT
GGAGGAGTTAGTTAAGAAAAGGGAGGAATGCTTAGATGCCTTGGAGTCAATCATGACAACAAAGTCCATAAG
TTTTAGACGCCTCAGCCATCTTCGGAAGTTGGTCCCTGGATTTGGGAAGGCATACACTCTGATCAACAAAACCC
TCATGGAGGCCGATGCTCACTACAAGTCAGTCAGGGAATGGACAGAGGTGATCCCCTCCAAAGGGTGTTTAA
AAGCCGGGGGAGGATGTTATCCCCATTATAACAGGGTGTTTTTCAACGGGATCATCCTGAGTCCTGATGGACA
TGTGCTAATTCCAGAAATGCAATCTGCTTTACTCCAGCAACATATTGAGCTGTTGGAATCTTCTGTCATACCCCT
CAGACACCCCTTGGCGGATCCATCTACTGTTTTCAAAGGGGATGATGAGGCGGAGGAGTTCGTAGAGGTCCA
TCTTCCGGACACGCAAAAGCAGATCTCGGGGATTGACCTAGGCCTTCCAGAGTGGaaaagaTACTTTCTAATGG
GGATGTCTGCCATCGGATTTCTTGCTTTGACGATAATCCTAGCGGTGTGCTGTCGGAGGATCAAGAGACGAAA
GCAGTCTAAACCCAACCCCGTTGAGCTGATTAAAAAGGTTTCTGTCACCTCTCAAAGTGGGAGAGCGATTCCA
TCATGGGAGTCTTACAAGGTCAAGACTGGGGATCAACCTCAAGTCTAG

>ABLV_RV634_Complete glycoprotein cds_P#7

ATGCTCCTTCAAATTGTCCTCCTTATGTCCCTGATGGTCTTCTCACCATGCCCTGGGAAATTCCCTCTCTACACA
ATACCCGACAAATTGGGCCCTTGGAGCCCGATAGATATTCATCATTTGAGCTGTCCAAACAACCTGATTGTTGA
GGATGAGGGATGTACAAGCCTGTCCGGGTTTTCATATATGGAGCTAAAGGTTGGGTTTATAACCACTATAAAG
GTTAGCGGGTTTACCTGCACTGGAGTTGTGACTGAATCAGAGACCTACACTAACTTCTTTGGGTATGTCACGA
CTACTTTTAAGAGGAAACACTTTCGTCCTACCCCGGAGTTTTGTAGGAATGCATACAACTGGAAAGTTGCAGG
TGACCCCAGGTATGAGGAGTCTTTGCACAACCCTTATCCAGACTATCACTGGTTGAGAACAGTTACAACCACTA
AGGAGTCTCTCTTGATCATATCACCCAGTGTAGTAGACATGGACCCCTATGACAAATCCTTGCATTCGAAAATG
TTCCCTAAAGGCACATGCTCGGGAGCCTCGGTCCCTTCGATTTTCTGCTCCACTAATCATGATTACACATTGTG
GATGCCTGAGAATCCTAAACCAGGAATGTCATGTGACATCTTTACAACAAGCAAAGGAAAGAAAGCATCGAA
GGGAGGAAAGGTGTGCGGTTTTGTTGATGAACGTGGATTGTACAAATCTCTGAAGGGAGCATGCAAACTTAA
GTTGTGCGGAATCTCTGGTCTCAGGCTTATGGACGGGTCTTGGGTTTCCATCCAAAATCATGAGGAGGCCAAA
TGGTGCTCTCCTGATCAGTTGGTAAATATTCATGATTTTCATTCAGATGAGATAGAACACTTAATTGTAGAGGA
ACTGGTCAGGAAGAGAGAAGAATGTTTGGATGCACTGGAGTCAATAATGACTACCAAATCAGTCAGTTTTAG
ACGTCTCAGCCACCTAAGGAAGCTTGTGCCAGGATTCGGGAAGGCTTATACCATTGTCAACAAAACACTCATG
GAGGCTGATGCACACTATAAATCAGTCAGAACATGGAATGAGATCATACCATCTAAAGGGTGTCTAAAGGTT
AGAGAGAGGTGTCATCCTCCTTATAACGGGGTGTTCTTTAACGGGATCATTTTGAGTCCAGACGGACATGTCT
TAATTCCGGAGATGCAGTCCTCCTTGCTACAGCAGCACATTGAGCTACTTGAATCATCCGTAATACCTCCTATC
CATCCTCTGGCAGATCCATCTACTGTCTTTAAAATAGATGATGAAGCAGAAGACTTCATAGAAGTCCACCTTCC
CGATGTCCAAAAGCAGGTCTCCGGGATCGACTTGGGTCTCTCGGAGTGGGAGAGATATCTGATCATCGGGGC
TTCTGCTGTAATCTTGTTTGCTCTGGCAATAATTTTTGCAGTTTGTTGCAGAAGGTGTAAGAGACGGAAAAAG

GCCAGAACTGATCGTATAGAGTTAGACCGTAAAGTGTCAGTAACATCTCAAAGTGGGAAAGTGATCCCTTCTT
GGGAATCCTACAAAATTGAAGCCGGAGGTCATTTCAGGTCGTGA

>DUVV_DUVVSA06_Complete glycoprotein cds_P#unknown

ATGCCACTCAATGCAGTCATCTTTACTCTTCTCCTCAGATGTTCCATCTGCCTTGGAAAGTTTCCCATTTATACG
ATCCCTGACAAATTGGGGCCATGGAGCCCTATAGACATACATCATCTCCGTTGCCCAAATAACTTGGTTGTGG
AAGACGAAGGATGTACAACTTTAACCCCTTTTTCATACATGGAGTTAAAGGTAGGTTACATTACATCAATAAA
GGTGTCCGGTTTTACCTGCACTGGGGTAGTGACGGAAGCTGAGACTTATACCAATTTCGTTGGGTATGTGACG
ACAACATTTAGAAGAAGACATTTCCGTCCATCAGTCAACTCTTGTAGAGATGCATACAATTGGAAGATCGCAG
GAGACCCTAGATATGAAGAATCACTACACAACCCTTATCCAGACTCTCACTGGCTCAGAACTGTCAAGACTACA
AAAGAATCTCTTTTGATAATCTCCCCCAGCGTGGCTGACATGGATGCTTATGACAAGAAGCTTTACTCCAAGAT
GTTTCCTAACGGAAGGTGTTCGGAAATATCTCCTGGGTCTCCCTTCTGTCCCACCAATCATGAATACACTATCT
GGATGCCCGAGAGCTCAAACCCCGGAATATCCTGTGACATATTCACAAGAAGCATGGGGAAGAAAGCCACCA
AAGATGGACAGTTATGTGGGTTTGTAGATGAGAGAGGACTGTACAAATCTTTGAAGGGAGCTTGTAGATTAA
GACTCTGTGGGATCAGTGGACTGAGACTGATGGACGGATCCTGGGTTTCACTCCCACAGGTTAACAACTCAGA
ATGGTGCTCCCCAGACCAACTCGTCAACATTCACGACTTCCATTCAGATGAGATAGAGCATCTTGTCGCAGATG
AGTTGGTAAAAAAGAGAGAGGATTGTCTAGACGCCCTTGAAACTATCATCTTCACCAAATCTATAAGTTTCCG
GCGGTTAAGCCATCTTCGGAAGCTGGTTCCAGGCTTTGGTAAAGCATATACTATCATAAATAGGACCCTTATG
GAAGCAGAGGCTCATTACAAGTCAGTGCGGGAGTGGAAGGAAATTATCCCATCCAAGGGGTGTCTGAAGGC
AGGAGGGAGGTGCTATCCTCATCACAATGGAATTTTCTTCAACGGGATCATTCTGGGTCCGGGAGGGGAGAT
CTTGATCCCTGAGATGCAGTCTGCCTTGCTCCAACAGCACATTGAGCTGTTAGAGTCCTCTGTAGTTCCCCTCA
ACACCCCCCTGGCAGACCCTTCGACTGTCTTCAAGAACGATGATGAAGCGGAGAGTTTCGTAGATGTTCACCT
TCCTGATACAAACCAAAAAATATCTGGAATTGACTTAGGGTTGCCAGAGTGGAAACGTTACTTCCTAATAGGA
GTCTCAGCAGTTGCTTTGTTGGCTTTATCTATAATTATCGCCGTCTGTTGCAAAAGGTTCAGGAAAAGGAGGA
AATCCAAACCCAGTCCAGTAGAATTGACTAGAAAAGTGTCAGTCATATCCAAAGGAAATGGACCGGTCCCTTC
CTGGGAATCTTATAAAGAAGGAACCACAGGAGATGTTCGTAATATGACTCCATCAACAAGGGAGTGA

>EBLV-1_RV20_Complete glycoprotein cds_P#11

ATGTTACTCTCTACCGCCATATTCGCCTTTTTCTTGAACTGTGCTCCTTGTCTGGCAAAGTTCCCAATCTACACCA
TCCCGGATAAGATTGGACCGTGGAGTCCTATTGACATAAACCATCTCAGCTGCCCGAACAACTTGATCGTGGA
AGACGAGGGATGCACGACTCTCACCCCGTTCTCGTACATGGAACTGAAAGTGGGATATATAACAACAATAAA
AATAGAAGGATTTACATGCACTGGTGTAATAACAGAGGCAGAGACCTACACAAACTTTGTAGGGTATGTGAC
TACAACCTTCAAGAGGAAACATTTCCGGCCAACTGTGAGCGCCTGTAGAGATGCCTACAACTGGAAGATTACC
GGTGATCCGAGATATGAAGAGTCACTGCACAATCCTTACCCTGACTCTCATTGGTTGAGAACTGTGAAGACCA
CCAAGGAGTCTCTTCTGATCATCTCTCCTAGTGTAGTCGACATGGACGCATATGACAAAAACCTCTACTCCAAA
ATGTTCCCTAATGGTAAATGTCTAGCTTCACCCCCGAGTGCCATATGTTGTCCGACAAATCATGATTACACCATT
TGGATTCCAGAGAATCCTAAACCCGGGCTGTCCTGTGATATATTCACAACTAGCAAAGGAAAGAAAGCAACCA
AAGACGGGAGGCTGTGTGGATTTGTGGATGAGAGAGGATTGTACAAATCCTTGAAGGGGGCTTGCAAGCAG
AGACTCTGTGGGGTCCCTGGCATGAGATTGATGGATGGATCATGGGTATCTCTTCAGAAAACTGAAGCTCCG
GAATGGTGCTCTCCAGATCAGCTTGTTAATGTCCATGATTTCCATACAGATGAAATTGAACACCTTGTGGTTGA
AGAGTTGGTGAAGAAGAGGGAAGAGTGTTTGGATGCTTTAGAAACTATCATCACAACCAAGTCTATAAGTTTC
AGACGTCTCAGTCATTTCAGGAAACTGGTTCCTGGGTTCGGAAAGGCATACACTCTCATCAACAAGACTCTCAT
GGAAGCAGATGCCCATTACAAGTCTGTAAGAGAATGGAAAGAAGTGATCCCATCTAAAGGGTGTCTGATGGC
AGGGGGGAGGTGCCATCCTCACTACAGTGGTATATTCTTCAATGGGATCATACTGAGTCCAGGGGGTGATGT

ACTCATTCCGGAGATGCAGTCAGCCTTGTTACAGCAGCACATTGAGCTGCTAGAATCCTCGATGATTCCTCTCA
GACATCCCTTGGCTGACCCTTCCACGGTGTTCAAGAGAGATGATGAGGCAGAGGATTTTGTTGAGGTTCACCT
TCCAGACACCCAAAAACTCATATCAGGCATAGATCTGGGTTTTCCCGAATGGAAGCGTTACTTTCTTATAGGAA
TATCTGTGCTAGCCCTTCTTGCCTTAGCAATAATTACTGCAGCATGTTGCAAAAGGTTCAAGAGGAGAAGGAG
GCCTAAGCCAAACCCAATAGAGTTGATCAGGAAAGTTTCTGTAACCTCTCAAAGTGGAAGGGCAATTCCATCT
TGGGAGTCGTACAAAGTGGGGGCCACTGGTGAGTCGTAA

>EBLV-2_RV628_Complete glycoprotein cds_P#8

ATGCCATTTCAAGCTGTCCTGTCTGCTCTTCTTTCGGCCCTCACACTCTGCGTCGGCAAGTTTCCCATCTACACA
ATCCCTGATAAGTTAGGCCCGTGGAGCCCCATTGACATTCATCATCTTAGCTGCCCGAACAATATTGTCGTAGA
GGATGAGGGATGTACGACCCTAACAGTATTCTCATACATGGAGTTGAAAGTAGGATATATAACAACCATAAAA
GTGAACGGATTTACATGTACTGGGGTGGTGACAGAGGCAGAGACTTATACTAACTTTGTTGGTTATGTAACTA
CCACGTTCAAGAGGAAGCATTTCCGTCCCAGCCCTAGTGCATGCAGAGATGCATACAGTTGGAAGACTGCAG
GTGATCCGAGATATGAGGAATCCTTACACAATCCCTATCCAGACTCTCACTGGCTAAGGACAGTTACCACCACC
AAAGAGTCCGTCCTGATAATCTCCCCCAGTGTGGCCGATATGGATGCGTATGACAAAACTCTTTATTCTAAAAT
ATTTTTAAATGGGAAATGCTCAGGAGTCTCTCAAGTCTCTCCCTTCTGCTCAACCAATCATGATTACACGATCTG
GATGCCCGAGAATCCCAACCCGGGTGTGTCTTGCGACATATTCACGACCAGTAAGGGGAAGAAAGCTACAAA
AGATGGCAAACTGTGCGGATTTGTCGATGAAAGAGGACTTTACAAATCATTAAAGGGGGCTTGTAAACTGAA
ACTGTGTGGAATCTCTGGTATGAGGTTGATGGATGGGTCATGGGTGTCTATTCAAAATCACGATGAGGCAAA
ATGGTGCTCCCCCGATCAACTTGTGAACATCCATGACTTTCATTCAGATGAGGTAGAACACCTCATTGCGGAG
GAGCTTGTTAAAAAGAGAGAGGAGTGTTTAGATGCGTTAGAGTCAATCATGACTACCAAGTCAATAAGTTTTC
GCCGTCTTAGTCATTTAAGAAAGCTAGTCCCTGGGTTTGGGAAAGCTTACACCATTATCAACAAAACACTCATG
GAAGCTGATGCTCATTACAAGTCTATTAGAGAATGGACCGATGTGATCCCCTCCAAGGGGTGTCTGATGGCAG
GAGGCCGATGTTATCCTCATCACAATGGAGTCTTCTTTAACGGTATAATATTGAGTCCAGATGGGCACGTGTTA
ATCCCAGAGATGCAGTCTGCAATGCTTCAACAGCATATAGAGTTGTTAGAGTCATCGGTGATACCTCTCATGC
ACCCCTTGGCTGACCCATCCACTATCTTTAAGAAGGATGACGGTGCTGAGGATTTTGTTGAAGTCCACCTTCCT
GACGTGCAAAAGCAGATCTCGGGGATTGACCTAGGTCTTCCCGAGTGGAAGAGGTATTTCCTGATCGGGGTG
TCGGCTTTGGCTTTCCTTGCCTTGATGATATTTATTGCCGCCTGCTGTAGGAGAGTTAAACGTAAGAAGAGAG
CCAAACCCAACCCAGTGGAACTAATCCGAAAAGTGTCTGTCACTTCCCAAAGTGGGAGACCGATTCCTTCATG
GGAGTCTTACAAGGTAGAGACGGGGGGTCAGTCTTAA

>IKOV_RV2508_Complete glycoprotein cds_P#8

ATGGCTCAGTTGGTCACTTTGGTGCTGAACGGATTGTCCCTTTTGACAGGGCCAGGCATATTTCCAATGTATAC
AATCCCTGAAGGGCTGGGTCCCTGGACTCCGATTGATTTATCTCATCTGAAGTGTCCTGACAATACATACTTTG
CAGAAGAGGGCTGCAATGAGGGCAGCAAGGTGTCTTACTTAGAACTGAAGCCTTCTTTTCATTCTCAAAACAA
AGTACAGGGATTTACCTGTACAGGGATAATCAACATGGCAACCACATACACCAATTTCGTTGGTTATGTCACC
ACCACTTTTCAGCGGTCCCACTTCATCCCCAACCAGAGGGATTGTCGTCAAGCTAGAGAGTGGAAGAAGGAA
GGGGACCCAAGATACGAAGAGTCATTGACCACTCCTTATCCCGACTCTAAATGGTTGAGAACAGTCACAACCT
CCAAGGAATCATGGCTGATATTGGATCCTGCAGTGGTTGAGATGGACATCTACAACAAGACAATGTTTTCTCC
AGTCCTTCGGAATGGATACTGCAACTTTTCTCCAGAAAATCCAGATTTTTGTGAGACAAATCATCAACACTCCA
TTTGGATTCCGGAAGATGAGGGACGAGGAATTACATGTGACATCTTCCAAGCGAGTACTGGTATATTACTGAA
AAATGGAAGCAAAGTCTGCGGTTTCCAAGATGAAAGAGGATTATTCAGAAGCATAAAGGGTGCATGCAAAAT
GATAATCTGTGGAAAGTCTGGGGTCCGACTATATGATGGGACATGGGTGTCTTACAATTCTGTTGATAACCTC
AGGATGTGTTCCAGATCAAAAATGGTGAACAAACACACAGTTAAATTGGATAATATAGAAGAGTCCATTGTGA

GAGATCTTATTAAGAAGCGTGAAGAGTGCCTAGATGCTCTAGAAGAAGTCATGTTGACTAGATCTATAAGCTT
CAGAAAGTTGAGTCTCTTTAGAAAGCAAGTTCCAGGAAGAGGGTATGTTTACACTATGATAAACAATACAATG
ATGGAGGCAACAGGGCATTATAAATCAGTGGATAACTGGACAGACATTCTACCTAACCCAATCTGCCTTATGG
TGGACGGCAAGTGTCATCCAGGTTATGATGGTGTATTATTTAACGGTATCATAAGAGACAGTAGAGGCAAAA
TACTAATTCCAGAAATGCAATCACATCTACTCCGAGACCATCTGGAGTTGCTCAAGAGAAATTCCATTCCCTGG
AGACACCCTCTTGTGCACTATTCTGAAAATGGGGAAGATGGCTCAGATCTTACCAGCTTTGCTCAGCTATACAT
AAAAGACCCTCATTTGTCTGTTTCTGACATAGATATAGGATTTCCTTCATGGAAGAAGTATTTGATGATTGTGG
GGGGAGTGTTGATAGGACTAATTGTCTTCTGGTTCTTGGTCAAGTTGTTGTTTAGAGTATGTTATACTGTTAAG
AGGAAAAGGCTTCGTGAGGGTTCTATGAATGAGACATCCAGTGGCCCTGTCATTCGGACTTCTGGTGACAAAC
AGCTTTCGTGGGAATCTTACAAGAGTTCTGCATTCTAG

>IRKV_RV3382_Complete glycoprotein cds_P#5

ATGTCGCTCCTCACTGCCGTTATTGCCTTTCTCTTCATTAGCACCTTCTGTTCAGGAAAATTTCCCATTTACACGA
TCCCCGACAAGATTGGGCCTTGGAGCCCCATTGATATAAATCATCTCAGCTGTCCGAACAACTTAGAGGTAGA
GGATGAGGGGTGTACTACTCTCACCGCATTCAACTACATGGAACTCAAGGTAGGATACATAACATCTATTAAA
GTCGACGGATTCACTTGTACAGGGGTGGTTACGGAGGCAGAGACTTACACGAACTTTGTGGGGTATGTGACC
ACAACATTTAAAAGGAAGCACTTTCGCCCTAATGTCAGCGCCTGCAGGGCAGCCTTTAGTTGGAAGACAGCAG
GCGATCCGAGGTATGAGGAGTCATTGCACAATCCCTATCCTGATTCCCATTGGTTGAGGACAGTAACCACCAC
AAAAGAATCTTTGTTGATTATCTCTCCTAGTGTTGTCGACATGGACGCCTATGACAAGACACTCTACTCCAAGA
TGTTCCCCAACGGGAAATGCTTTCCACCAATATCAGATTCTCCATTCTGCTCGACCAATCATGATTATACATTAT
GGCTTCCCGAAAAAGAGAAGCTTAGCATGTCCTGCAATATATTTGTCTCCAGTAAGGGGAAGAAAGCAACAA
AGGACGGTAGATTGTGCGGATTTGTCGATGAAAGAGGACTGTACAAGTCTCTCAAAGGGGCATGTAAATTGA
AGTTATGTGGGATGGCAGGCATGAGGCTGATGGACGGGTCTTGGGTGTCCCTACAAAGGGCAGATGCCCCTG
AGTGGTGTCCCCCCGGTGCTCTTGTTAATGTGCACGATTTTCATTCAGACGAGATAGCCCACTTTGTTGTTGAG
GAGCTAATAAAGAAACGAGAAGAATGCTTGGACACCTTAGAGACCATCCTCACCACTAAGTCTATAAGCTTTA
GGCGTCTGAGCCATTTCAGAAAGCTGGTGCCTGGTCTAGGGAAGGCATATACTCTAATAAACAACACCTTGAT
GGAAGCTGAAGCTCACTATAAGTCTATTAGGGAATGGAAAGAAATTATTCCATCTAAAGGTTGTTTGAAGGCG
GGGGGGCGTTGTCATCCTCACTATGATGGGATCTTTTTCAATGGGATAATTCTCGGTCCGAACGGAGATGTGC
TCATCCCAGAGATGCAATCGTCCCTGCTTCAGCAACACATAGAGTTGTTGGAGTCATCTATGATTCCTCTTAGA
CACCCTCTGGCTGATTCTTCTGCTATATTCAGGAGTGATAATGAGGCTGAGGACTTTGTAGATGTTCATCTTCC
TGATACACAAAAACAAGTGTCAGATATAGATTTGGGCTTTCCGGAATGGAAGCGCTATTTCTTGATAGGGGTC
TCTGCAATTGCCTTGTTCTCTCTAGCAATAATAATAGCTGTTTGTTGTAGAAAGTTCAAGAGAAGGAAGAGGC
CCAAACCAGGCCCAATTGAGTTGGTGAGGAAAGTTTCCGTTACTTCCCAAAGTGGAAAGGTGGTCCCGTCTTG
GGAGTCCTACAAAGAGGGTGCCACAAGTCAACCTTAA

>KHUV_RV3380_complete glycoprotein cds_P#8

ATGCCCTCTCAAGCAGTCTTTCTTGTTCTTACCACGGTTTTCTCTCAGTGTGTTGGGAAATTCCCTATTTACACTA
TTCCTGACAAATTAGGACCTTGGAGCCCCATAGACATTCATCATCTTAGCTGTCCGAATAATTTAGTGGTGGAA
GATGATGGGTGCACCACCCTATCTGGGTTCACGTATATGGAGTTGAAGGTGGGATATATAACAACGATAAAA
GTAGATGGTTTTACATGTACTGGAATAGTGACAGAGGCAGAAACCTACACAAATTTTGTTGGTTACGTTACCA
CCACTTTCAAAAGGAAACATTTCAGGCCAGGCCCAAGTGCATGCAGGGATGCATACAACTGGAAAGCCGCAG
GTGACCCTAGATACGAAGAGTCCTTGCACAATCCATATCCAGATTCTCACTGGTTGAGAACCGTCACAACCACT
AAAGAGTCTCTTTTAATCATTTCGCCTAGCGTGGTGGACATGGATGCATACGACAAATCTCTTCTCTCTAAGAT
ATTCCCTAACGGGAAGTGCCCCGGGGTATCCATTGCCTCTCCATTTTGCTCCACCAATCATGATTACACAATTTG

GATGCCTGAAAACACAAAGACCGGAATGTCGTGTGATATATTCACGACCAGTAAAGGGAAGAGAGCTACTAA
GGATGGTAAGTTGTGTGGGTTTGTTGATGAGAGGGGGCTCTATAAATCATTGAAGGGATCATGCAAATTGAA
ACTGTGCGGTGTCTCTGGCTTAAGGCTGATGGACGGGTCATGGGTCTCCATCCAAAATCACGAAGAGGCCAA
GTGGTGCCCTCCCGATCAACTGGTGAACGTTCATGATTTCCACTCAGATGAGATCGAGCATCTGATTGTAGAG
GAGCTTGTTAAGAAGCGGGAGGAGTGTTTGGACGCCTTAGAATCGATCATGACAACCAAATCGATCAGTTTTC
GTCGCCTCAGCCACCTGAGAAAACTTGTTCCTGGGTTTGGCAAAGCTTACACCATCATAAACAAGACACTCAT
GGAAGCTGATGCTCATTACAAGTCGATAAGGGAATGGTCAGAAATTATCCCATCCAAGGGATGTTTGGTAGC
GGGAGGCAGATGTTATCATCATCACAATGGAGTATTCTTCAACGGGATAATCTTGAGCCCTGATGGACATGTA
TTAATCCCGGAGATGCAGTCTGCTTTGCTTCAACAACATATCGAGCTATTGGAGTCATCTGTAATACCACTCAT
GCaTCCACTGGcTGACCCTTCCACTGTTTTCAAAGGGGATGATGGGGcGGAGGATTTTGtAGAGGTTCATCTTC
CaGATGTTCAAAAACAGATCTCTGGAATTGATTTAGGCCTTCCGGAGTGGAAGAGATACTTCTTAATAGGAGT
GGCTGCATTGACCCTATTTGCCTTGACAATATTTGTTGTGGTGTGCTGTCGAAGAGTTAGGAGGAGGGAGAG
GGCCAAACCCAATCCAGTGGAATtGATTCGAAAGGTGTCTGTGACTTCTCAAAGCGGCAAAGTGATCCCTTCA
TGGGAATCATACAAGGTGGAGGCAGAGGGTCAATCCCAAGCCTAA

>LBV A_LBVAFR1999_Complete glycoprotein cds_P#unknown

ATGAGTCAATTGTTCTCAACCTTCATCTTTTGCTTGTTCATAGGAAGTTCCATAGGAGACTTCCCTCTATATACA
ATTCCCGAACGGCTTAATCCCTGGACTCCAATTGACTTAATCCACCTGAGCTGTCCTAACAACTTACTGTCTGAT
GCTGAGGGGTGCAGTGAAACTTCATCCTTTACCTATGTAGAGCTCAAAACCGGTTTTCTTGCCCATCAGAAAGT
GCCGGGGTTCACTTGCACAGGTGTGATAAACGAGGCAGTCACATACACCAACTTTGTTGGATATGTTACTACC
ACATTTAAGAGAAAACATTTTAAACCAACTGTTTCTGCATGTAGGGATGCCTTCAACTGGAAAGTTTCTGGAG
ATCCTAGATACGAGGAGTCTTTACATACACCCTATCCGGATAACAGTTGGCTGCGAACTGTCACCACAACCAA
AGAATCCTTGTTAATAATCTCTCCAAGCATTGAAGAGATGGATGTATACGGTAGAACTCTCCATTCCCCCATGT
TTCCTACAGGAATCTGCTCTAAGTTTTATCCATCAATTCCCTCATGTGCTACCAATCATGATTACACGTTGTGGT
TGCCTGAAGACCCTAACTTGAGTTTGATTTGTGACATATTTGTGACAAGCACAGGAAGGAAGGCAATGAATG
GGTCTCGTATGTGTGGATTCACAGATGAACGAGGGTTTTATCTAACCCTGAAGGGAGCATGCAAGTTGACCTT
GTGTGGGAAACCAGGCCTACTGTTATATGATGGAACATGGGTCTCGTTCACTAGGCCTGAAATCCATGTATGG
TGCTCTCCTGATCAGCTGGTGAACGTGCATAACAACCGCATAGACGAAATTGAACATTTGATTGTAGATGACC
TCATCAGGAAGAGAGAGGAGTGTTTGGATACACTAGACACTGTTCTTATGTCTAAGTCGTTAAGTTTCAGAAG
GCTCAGTCACTTCCGGAAGTTAGTCCCTGGGTATGGAAAGGCCTATACAATCCTCAATGGGAGTCTGATGGAA
ACCAATGTTCACTATAAAAGGGTGGATAATTGGGTCGACATACTGCCTTCCAAAGGGTGCCTTAAGGGAAACA
ATAAATGTATGGAGTCATGCACACGGGTTTTCTTCAATGGAATCATGAAAGGGGCCGATGGTAGGATTCTCAT
CCCTGAAATGCAGTTTGGACTGGTAAAACCACATAAGGGCCTCTTGAAGGCTGCAGTGTTCCCTCTAAGACAC
CCATTGATTGACAAAGCCTCAGTGTTCAAAAAGGATGGTGATGCAGACGACTTCGTCGATGTCCACATGCCAG
ACATCCAGAAGCTAGTATCTGATGTAGACTTGGGCTTGCCAAATTGGAGTTTCTTTGCTTTGATAGGTGCCAGT
GTAATTGCACTGCTCATTCTAGTATGTCTTCTGAGGATGTGCTGCAAACGGATGAGGAGAAGAAGCTCTGTCA
GGGATCAAGTAGCCCCACCGTTTAGCTTATCTTCTACTCCTTCTTCTAAGTCAAAGGTGGTGTCATCATGGGAG
TCATATAAAGAGACATCAAGTGTCTAA

>LBV C_UP6414_compete glycoprotein CDS_P#17

ATGAGTCAGCTAATTCTGATACCATTTTTGTGTGTGGTTATAGTGACATCAGTGGGAGATTTTCCTTTATATACT
ATCCCGGAAAAGATAGGCACCTGGACTCCAATCGATCTGATTCATCTAACTTGTCCTAACAATCTGCTGTCAGG
GGATGATGGGTGCAGTGATACTGCCACTTTTAGTTATATAGAGCTTAAAACAGGTTACCTCACTCATCAAAAA
GTTTCTGGCTTTACATGCACAGGTGTAGTTAATGAGGCTGTCACTTACACCAACTTTGTAGGATATGTGACCAC

CACCTTCAAAAGGAAGCATTTCAAACCCACAGCCTTAGCGTGTAGAGACGCCTTTCATTGGAAAATCTCTGGA
GACCCGAGATATGAGGAGTCTTTGCACACACCATACCCTGACAATAGCTGGTTAAGAACAGTTACTACAACGA
AGGAGTCCTTGCTAATAATCTCCCCCAGTATAGTTGAGATGGACGTTTATAGCAGAACCCTTCACTCTCCTATG
TTTCCTGGTGGGGTATGCTCTAAATTCTATCCATCATCCCCTTCCTGTCCTACTAATCATGATTACACCCTATGG
CTTCCTGAGGATGCCAATCTAAGTATGGCCTGTGATATATTTGTTACCAGTACAGGGAAAAAGTCAATGAATG
GTTCAAGAATGTGTGGATTCACTGATGAAAGAGGATTCTACAGGACACTAAAAGGGGCATGTAAGCTGACTT
TGTGCGGGAAGCCGGGATTGAGGTTATATGATGGAACATGGGTATCTTTCACACGCCCAGAGATTAACGTAT
GGTGTTCTCCCAACCAACTAGTCAATGTTCATAACAATCGATTGGATGAGATAGAACATCTAATTGTGGGAGA
CCTCATTCGGAAGAGAGAAGAGTGTCTGGACACTCTAGAGACCATTCTGATGTCCAAATCTATAAGCTTTAGG
CGACTCAGTCATTTCAGAAAGTTGGTGCCTGGGTATGGGAAAGCTTATACAATTATTAACGGGAGTCTGATGG
AAACCAATGTTCACTACCTTAGGGTTGACAGTTGGAATGATATATTGCCTTCCAAAGGATGCTTGAAAATGAA
CAAACAATGTGTCGATTCCTACAAAGGAGTCTTCTTCAATGGGATTATCAAAGGACTGGATGGACATATTTTG
ATTCCAGAGATGCAGTCCAGTCTACTGAAACAGCATATGGATCTCTTAAAGGCAGCAGTTTTTCCTCTACGACA
CCCCTTAATAGACCAAAATTCTTTGTTTAAGAAGGATGGTGATGCAGATGATTTTGTGGAAGTTCACATGCCA
GATATACAAAAGTTKATCTCTGATGTAGACCTCGGATTGCCAAGTTGGAGTCTCTACGTCCTGATAGGAGCTG
CTGTAATAGCTTTCCTGATCCTGATATGCTTAATCCACATTTGCTGCAAGAAGAGGGGTCGCACGCGCCCCTCG
ATAGAAAGACCAGATCCTCCCCTAAGCTTGTCCACTACTCCCCAGTCTCGGGCTAAAGTAGTGTCGTCTTGGGA
GTCCTACAAGGGAACTTCAAATGTGTAG

>LBV D_KE576_Complete glycoprotein cds_P#4

ATGAGTTACTCGATTTCAACACCTTTTCTGTTCGCTGTGGCTGCAATTTCAGTTGCAGACTTCCCCCTTTACACA
ATTCCCGAGAAGATCGACTCCTGGACGCCTATAGATATGATCCATCTGAGTTGTCCAAATAACCTTTTGTCAGG
GGATGACGGGTGCAGCACATCCACATCCTTCAGTTACGTGGAGCTCAAAACAGGTTATCTGACTCATCAGAAA
GTGTCTGGGTTCACCTGTACCGGGGTCATCAGTGAGGCTGTTACATACACAAACTTTGTAGGTTATGTGACAA
CCACATTTAAAAGGAAGCATTTCAAGCCCACGGTCTCAGCCTGTAGAGATGCATTTCATTGGAAGATCTCAGG
GGATCCTAGATATGAAGAGTCTCTGCATACGCCTTACCCTGACAGTAGTTGGTTAAGAACGGTCACCACAACA
AAGGAATCCTTGCTGATAATCTCCCCCAGCATTGTAGAGATGGATATATATAGTAGAACTCTTCACTCTCCTAT
GTTTCCCACAGGAACTTGTTCCAGATTTTATCCATCAGTTCCATCCTGCGCTACCAATCACGATTACACCTTGTG
GTTGCCAGAAGATTCTAACTTAAGTCTCATTTGTGACATATTCGTTACTAGCACAGGTAGAAAAGCAATGAAC
GGCTCCAAGATGTGTGGGTTCACAGATGAGAGGGGTTTTTACAGAACACTGAGAGGGGCCTGTAAATTGACG
CTCTGCGGGAAACCAGGTCTAAGACTATATGATGGAACATGGGTATCCTTTACACGACCGGAAGTTCATGTAT
GGTGCTCCCCGAATCAACTAGTCAATGTGCATAATAACCGTATCGATGAGCTTGAGCATCTGATTGTGGATGA
CCTTATCCAAAAAAGAGAAGCATGTTTAGATACCCTGGAAACAATTCTGATGTCTAAGTCAATTAGTTTCAGGA
GACTCAGTCATTTCAGAAAGTTGGTACCTGGATATGGAAAGGCTTACACCATATTAAACGGGAGCTTGATGGA
GACAAATGTCCACTACAGAAGGGTGGACAATTGGACTGATATCTTACCATCGAAAGGATGTCTGAAAGTGGG
GAACAAATGCCTTGATCCCCATAAAGGAGTTTATTTCAATGGTATTATTAAGGGTCCGGATGGTCATATTTTAA
TCCCAGAGATGCAGTCGGGCATGCTGAAACAGCATATAGATCTTCTGAAGGCAGCGGTTTTTCCCCTCCGTCA
CCCTCTTATAGATCAGAGTTCTGTGTTCAAAAAAGATGGTGATGCAGATGATTTTGTTGATGTTCACATGCCGG
ATGTAAACAAGTTAGTATCGGACATAGATCTAGGGTTCCATCACTGGGGTCTGTTCATTCTGATAGGGGTGAC
AGTATTGGCGTTTCTTGTTCTGATATGTCTTCTTTGTATTTGTTGTAAGAGAGTGAAGAGGGGAAGGTCTACTG
GGGATAGGACGGACCCGTCATTAGCATTATCTTCAATCTCTCAATCAAAACCGAAAGTGGTGTCATCTTGGGA
GTCTTACAAGGGCTCCTCAAATGCCTAA

>MOKV_14/024_Complete glycoprotein cds_P#12

ATGAATGTACCCTGCCTTGCTATAATCCTTGGCTTCACAACTACATATTCTCTTGGGGAATTTCCTTTGTACACA
ATTCCCGAGAAGATAGAGAAATGGACCCCAATCGATATGATCCATCTAAGCTGCCCCAACAACTTGTTATCCG
AGGAGGAAGGTTGCAATACAGAGTCGCCCCTCACCTACTTCGAGCTCAAGAGTGGTTACTTAGCTCACCAGAA
AGTTCCGGGGTTTACCTGTACAGGGGTAGTGAATGAGGCGGAGACATACACAAATTTTGTCGGGTATGTCAC
CACAACCTTCAAAAGAAAACACTTTAGGCCTACAGTCTCCGCCTGTCGTGATGCCTACAACTGGAAAGTGTCC
GGGGACCCCAGGTATGAGGAGTCACTGCACACTCCTTACCCTGACAGCAGCTGGTTGAGGACTGTAACCACC
ACCAAAGAATCCCTTCTTATAATATCGCCTAGCATCGTGGAGATGGATGTATATGGTAGGACTCTCCATTCCCC
CATGTTCCCTTCAGGAATATGTTCTAAGCTCTATCCCTCTGTACCATCCTGTAAAACCAACCATGATTACACATT
ATGGCTGCCAGAAGATCCTAGTTTGAGCTTAATCTGTGATATCTTCACTTCTGGCAGCGGAAGGATGGCCATG
AATGGGTCCCGCATCTGCGGATTCAAGGATGAAAGGGGATTTTACAGATCTTTGAAAGGCGCTTGTAAGCTG
ACATTGTGCGGGAAGCCTGGGATCAGATTATTTGACGGAACTTGGGTCTCTTTTACAAGGCCAGAAGTTCACG
TGTGGTGCACCCCTAACCAATTGGTCAATATACACAATGATAGAATAGATGAGATCGAGCACCTGATTGTTGA
AGACATTGTCAAAAGAAGAGAGGAGTGTTTAGACACTCTAGAGACAGTATTCATGTCTCAATCAATTAGTTTT
AGGAGGTTGAGCCACTTTCGGAAATTGGTTCCGGGGTATGGGAAAGCTTACACCATTTTGAATGGTAGCCTG
ATGGAAGCAAATGTCTACTATAAAAGAGTTGACAGGTGGGCGGACATTTTACCCTCTAAGGGATGTCTGAAA
GTCGGGCAACAATGTATGGACCCTGTCAACGGAGTCCTCTTCAATGGGATTATCAAAGGTCCGGATGGCCAG
ATCCTGATCCCTGAAATGCAGTCAGAGCAGCTCAAGCAGCATATGGACTTATTAAAGGCAGCAGTGTTCCCTC
TCAGACATCCTTTAATCAGCCAAAACGCCGTCTTTAAGAAAGACGGAGAGGCAGATGATTTTGTGGACCTCCA
TATGCCAGATGTGCACAAATCTGTATCAGATGTCGACTTGGGTTTGCCTCACTGGGGGTTCTGGCTGTTGATC
GGGGCAACTGTAGTGGCATTTTTGGTCTTGGTGTGTCTGCTCCGTGTTTGCTGTAAGAGAGTGAGGAGGGGA
GGTTCACGACGTACAACTCAGGAGATCCCCCTCAGCGTTTCCTCTGTCTCCGTCCCTCGGGCTACAGTGGTGTC
ATCATGGGAGTCTTATAAAGGGCTCCCAGGCACCTGA

>MBLV_UP5619_Complete glycoprotein cds_P#15

ATGGCTTCCTATTTTGCATTGATCCTGAACGGAATCTCTGTGGTTTTCAGTCAGGGACTTTTCCCCCTTTACACC
ATTCCTGATCATCTGGGGCCATGGACCCCGATAGATCTGAGTCATCTTCATTGTCCTGACAATCTTTATTCTGAT
GCTTCCGACTGCACCTCTGAACAAAGCATCACTTATACAGAGTTGAAGGTCGGATCATCAGTTTCACAAAAAA
TTCCCGGCTTCACCTGTACAGGAGTAAGGACGGAAGCTATCACCTACACTAATTTCGTCGGATATGTGACCAC
AACCTTCAAGAAAAAACATTTCTCTCCGAAACCCCGAGATTGCCGAGAAGCATATGAAAGGAAGAAGTCGGG
AGACCCTCGATACGAGGAGTCCTTAAATCATCCGTATCCAGACAACAGCTGGCTTCGAACAGTGACAACGACG
AAAGACTCTTGGGTGATAATTGAACCTAGCGTGGTTGAGCTAGACATTTACACAAGTGCCTTGTACTCACCCTT
TTTCAGAGATGGAATATGCTCTAAGGCTAGAACTTATTCTCCCCATTGTACTACTAATCATGACTTCACTTTGTG
GATGCCTGAGGAGGAGAATATACGATCTGCTTGTAACCTGTTCTCTACCAGTCGAGGAAAGTTAGTTCGAAAT
AGAACATCTACTTGTGGAATAATCGATGAAAGGGGGCTGTTTAGGTCTGTCAAGGGGGGCTTGCAAAATATCC
ATCTGTGGTAGACAAGGAATCCGGTTGATTGATGGAACATGGATGTCTTTCAGATATTCTGAATACCTGCCTG
CTTGTGCGGTATCGCAGTTAGTTAATACTCACGACATTAAAGTTGACGAGTTGGAAAGTGCCAATTGTTATGA
CCTCATTAGAAAAAGAGAAGAATGTCTTGATACTCTCGAGACTATCTTAATGTCTGGATCAGTCAGTCACAGA
AGGCTTAGCCATTTCAGAAAGCTGGTTCCAGGGTCTGGTAAAGCCTACTCTTATATAAACGGAACCTTAATGG
AATCTGACGCTCATTACATTAAAGTTGAAAACTGGTCAGACATCATTCCATATAAGGGGTGCCTCAGGGTAGG
AGGGAAGTGTTATGAACCGGTTAACGGTGTGTATTTCAACGGAATTATCAGAGATTCCAACAATCAAGTACTG
ATCCCAGAGATGCAATCAAGTCTCCTCAGAGAGCATGTCGACTTGTTAAAAGCAAATATTGTTCCGTTCAGGC
ATCCAATGTTGATCAAATCTTTCACCTCAGACAATGATGAGGAGATTGCCGAGTTTGTTAACCCCCATCTTCAG
GACACTCAGAAGTTGGTGTCTGACATGGATCTCGGATTGTCCAACTGGAAAAGATATCTCTTAATAGGATCCT
TAGCAGTGGCAGGGATCATTGTCTTTTTGTTTCTTGGGACTTGTTGTTTGAGATGCCGGTCAAGAGGAAGACG

TGGTATGAATCATTCAAAGCAAAAGTCCCCATCTCTGGATGTTATATTCCACAAGGATAAAGACAAAGTGATC
ACATCTTGGGAATCCTACAAGGGAAGCAATGTTCACTGA

>RABV_CVS-11_Complete glycoprotein cds_P#unknown

ATGGTTCCTCAGGTTCTTTTGTTTGTACCCCTTCTGGGTTTTTCGTTGTGTTTCGGGAAGTTCCCCATTTACACG
ATACCAGACGAACTTGGTCCCTGGAGCCCTATTGACATACACCATCTCAGCTGTCCAAATAACCTGGTTGTGGA
GGATGAAGGATGTACCAACCTGTCCGAGTTCTCCTACATGGAACTCAAAGTGGGATACATCTCAGCCATCAAA
GTGAACGGGTTCACTTGCACAGGTGTTGTGACAGAGGCAGAGACCTACACCAACTTTGTTGGTTATGTCACAA
CCACATTCAAGAGAAAGCATTTCCGCCCCACCCCAGACGCATGTAGAGCCGCGTATAACTGGAAGATGGCCG
GTGACCCCAGATATGAAGAGTCCCTACACAATCCATACCCCGACTACCACTGGCTTCGAACTGTAAGAACCAC
CAAAGAGTCCCTCATTATCATATCCCCAAGTGTGACAGATTTGGACCCATATGACAAATCCCTTCACTCAAGGG
TCTTCCCTGGCGGAAAGTGCTCAGGAATAACGGTGTCCTCTACCTACTGCTCAACTAACCATGATTACACCATT
TGGATGCCCGAGGATCCGAGACCAAGGACACCTTGTAACATTTTTACCAATAGCAGAGGGAAGAGAGCATCC
AAAGGGAACAAGACTTGCGGCTTTGTGGATGAAAGAGGCCTGTATAAGTCTCTAAAAGGAGCATGCAGGCTC
AAGTTATGTGGAGTTCTTGGACTTAGACTTATGGATGGAACATGGGTCGCGATGCAAACATCAGATGAGACC
AAATGGTGCCCTCCAGATCAGTTGGTGAATTTGCACGACTTTCACTCAGACGAGATTGAGCATCTCGTTGTGG
AGGAGTTAGTCAAGAAAAGAGAGGAATGTCTGGATGCATTAGAGTCCATCATGACCACCAAGTCAGTAAGTT
TCAGACGTCTCAGTCACCTGAGAAAACTTGTCCCAGGGTTTGGAAAAGCATATACCATATTCAACAAAACCTT
GATGGAGGCTGATGCTCACTACAAGTCAGTCCGGACCTGGAATGAGATCATCCCCTCAAAAGGGTGTTTGAA
AGTTGGAGGAAGGTGCCATCCTCATGTGAACGGGGTGTTTTTCAATGGTATAATATTAGGGCCTGACGGCCAT
GTCCTAATCCCAGAGACGCAAGCATCCCTCCTCCAGCAACATATGGAGTTGTTGAAATCTTCAGTTATCCCCCT
GATGCACCCCCTGGCAGACCCTTCTACAGTTTTCAAAGAAGGTGATGAGGCTGAGGATTTTGTTGAAGTTCAC
CTCCCCGATGTGTACAAACGGATCTCAGGGGTTGACCTGGGTCTCCCGAACTGGGGAAAGTATGTATTGATGA
CTGCAGGGGCCATGATTGGCCTGGTGTTGATATTTTCCCTAATGACATGGTGCAGAAGAGCCAATCGACCAGA
ATCGAAACAACGCAGTTTTGGAGGGACAGGGAGGAATGTGTCAGTCACTTCCCAAAGCGGAAAAGTCATACC
TTCATGGGAATCATATAAGAGTGGAGGTGAGATCAGACTGTGA

>RABV_*Desmodus rotandus* strain_Complete glycoprotein cds_P#7

ATGATCCTTCAGGCCCTTCTGTTTGTGCCTCTCCTAATCTCTTCGTTGTGTCTCGGGAAATTCCCCATCTACACA
ATACCGGACAAGCTCGGTCCTTGGAGCCCCATCGATATACATCACCTCAGCTGTCCAAATAATTTAGTTGTGGA
GGACGAGGGTTGCACCAATCTATCAGGGTTCTCTTACATGGAACTAAAGGTGGGATACATCTCTGCCATAAAG
GTGAACGGGTTCACTTGTACCGGTGTTGTGACAGAGGCTGAAACCTATACCAACTTTGTTGGTTATGTCACCA
CTACATTCAAGAGGAAACATTTCCGCCCTATACCGGATGCATGCAGGGCTGCATACAACTGGAAGATGGCTG
GTGATCCTAGATATGAGGAGTCTCTACAAAATCCTTATCCTGATTACCACTGGCTACGGACTGTAAAGACCACT
AAGGAGTCTCTTATCATCATATCCCCGAGTGTGGCTGATTTAGACCCATACAACAAATCCCTTCATTCTAGGGT
GTTCCCTGGTGGGAAGTGTTTGGGAATAACGGTTTCTTCCACCTACTGCTCAACCAACCATGATTACACCATAT
GGATGCCCGAGGAACCAAGACTCGGGACATCTTGCGACATTTTTACCAGCAGCAAGGGGAAAAAGGCATCTA
AGGGAGGCAAGACTTGTGGATTTGTGGATGAAAGGGGCTTGTACAAGTCTCTAAAAGGAGCATGTAAACTCA
AGCTGTGCGGAGTTCTCGGACTTAGACTTATGGATGGAACCTGGGTCTCAATTCAAACATCAGACGATACCAA
ATGGTGCCCTCCGGATCAATTGGTGAATCTACATGATTTTCACTCAGACGAAATAGAGCATCTCGTCGTGGAG
GAGTTGGTCAAGAAGAGGGAAGAGTGTTTGGACGCATTGGAGTCCATCATGACCACCAAATCTGTAAGTTTT
AGACGTCTCAGCTATTTGAGAAAACTTGTCCCTGGGTTTGGAAAGGCATACACTATATTCAACAAGACTTTGAT
GGAGGCTGACGCCCACTACAAGTCAGTTCGGACTTGGAACGAGATCATCCCCTCAAAAGGGTGTTTGAAAGT
CAGAGAGAGGTGTCATCCTCCTGTGGACGGAGTGTTCTTCAATGGCATAATTCTGGGTCCGGACGGGAATGT

CCTGATACCAGAGATGCAATCGTCTCTACTTCAACAACATATGGAGCTGTTGGAGTCTTCTGTAATCCCCTTAA
TGCATCCCTTGGCGGACCCGTCAACAGTCTTCAAGGAAGGGGATGAAGCGGAGGATTTTGTTGAAGTTCACCT
CCCAGATGTTCACAAACAGATCTCAGGGGTTGACCTTGGTCTCCCGAGTTGGGGGAAATATCTCCTGATGATT
GCAGGTGGTCTGGCGACACTAGTTCTGATAATCTGCTCGATGGCATGCTGTAGAAGAACCAAGCGAACAGAG
TCGAGAAGGCGAGGCTCTGTAGAGTCAGGGAAAAAGGTAACGGCAACCCCCCAGACTAGGAAAGTCGTATC
TTCATGGGAGTTGTACAAGAGTGAAGGCGATGCCAGGCTGTGA

>RABV_*Eptesicus fuscus* strain_2401_Complete glycoprotein cds_P#12

ATGATCCCTCAGGCTCTTCTGTATGTGCCTCTCCTGATCTCCTCGTTGTGTTTCGGGGGATTCCCCATCTACACG
ATACCAGACAAGCTCGGCCCCTGGAGTCCCATTGACATACATCATCTCAGCTGTCCCAACAATTTGGTTGTGGA
GGACGAGGGGTGCACCAGTCTGTCAGGGTTTTCTTACATGGAACTGAAGGTGGGGTACATCTCTGCCATAAA
GGTGAATGGGTTCACTTGTACTGGTGTCGTGACAGAAGCCGAGACCTACACCAACTTTGTtGGTTATGTCACC
ACTACATTCAAGAGAAAACACTTTCGCCcTATGCCAGACGCATGCAGAGCTGCATACAATTGGAAGATGGCCG
GTGATCCTAGATATGAAGAATCTCTTCAAAATCCTTATCCTGACTACAGTTGGCTACGGACCGTAAAGACCACC
AAAGAGTCTCTTATTATCATATCTCCAAGTGTAGCGGATTTGGACCCATACGACAAAtcCCtTCATTCAAGAGTT
TTCCCTGGTGGGAAGTGTTTGGGAATAACAATCTCTTCTACCTACTGCTCAACTAACCATGATTACACCATCTG
GATGCCTGAAGAAGCAAGACTCGGGACTTCTTGTGACATTTTTACCAACAGCAGAGGGAAGAGAGCATCCAA
GGGGGGCAAGACCTGCGGATTCGTGGACgAAAGGGGCTTGTACAAGTCTCTAAAAGGAGCATGTAAACTTAA
GCTGTGCGGAGTTCCCGGACTCAGACTTATGGATGGAACCTGGGTCACAATTCAGACATCAaATGAAACCAAA
TGGTGCCCTTCTGATCAATTGGTAAATCTACATGACTTTCACTCAGATGAGATAGAACATCTTGTCGTGGAGGA
GTTGGTCAAGAAGAGGGAAGAATGTCTGGATGCACTGGAGTCCATCATGACCACCAAATCCGTAAGCTTCAG
ACGTCTTAGCCACTTGAGAAAACTTGTTCCTGGGTTTGGAAAGGCATATACTATATTCAACAAGACCTTAATGG
AAGCTGAtGCTCATTACAAGTCAATTCGGACCTGGAACGAAATCATCCCCTCAAAaGGGTGCCTAAAAGTCGG
AGGGAGGTGTCaTCCTCATGTGGACGGAGTGTTCTTCAATGGTATAATTCTGGGTCCGGACGGGAATGTCCTG
ATCCCTGAGATGCAATCGTCCCTACTTCAACAACATGTGGAGCTGTTGGAGTCCTCTGTAATCCCTCTAATGCA
TCCCTTGGCAGACCCATCAACAGTCTTTAAGGACGGTgATGAAGCGGAGGACTTTGTTGAGGTTCACCTTCCG
GATATTCACAAACAGGTCTCAGGGGTCGACCTTGGTCTCCCAAACTGGGGGAAGTATCTGCTAATGAGTGCA
GGTGCTCTGGCAACACTAATGCTGATAATCTTCTTAATGGCATGTTGCAGAAGAGCTAATCGAACAGAATCAA
TACAACGAGATCCCGGGGAGTCAGGAAGGAAAGTATCGATACCTTCCCAAGACGGGAAAGTCATATCTTCAT
GGGAGTTGTACAAGAGTGGAGGCGAGACCAGGCTGTGA

>RABV_*Tadarida brasiliensis* strain_FL385_Complete glycoprotein cds_P#8
ATGATCTTTCAGGCTCTTCTATTTGTGCCTCTCCTAATTTCTTCGTTGTGTCTTGGGAAATTCCCCATCTACACGA
TACCAGACAAGCTCGGTCCTTGGAGCCCCATCGACATACATCACCTCCGCTGTCCAAACAATTTGGTTGTGGA
GGACGAGGGGTGCACCAGCCTGTCAGGGTTTTCTTACATGGAACTAAAGGTGGGATACATCTCTGCCATAAA
GATGAACGGGTTCACCTGTACCGGTGTTGTGACAGAAGCTGAGACCTATACTAACTTTGTTGGTTATGTCACC
ACCACATTCAAGAGGAAACATTTTCGCCCTATACCAGATGCATGTAGGGCTGCATACAACTGGAAGATAGCTG
GTGATCCCAGATACGAGGAGTCTCTTCAAAATCCTTATCCTGATTATCATTGGCTACGGACAGTGAAGACCACC
AAGGAGTCTCTTATCATCATATCTCCAAGTGTGGCTGATTTGGACCCATATGACAAATCCCTTCATTCTAGGTT
GTTCCCTGGTGGGAAGTGTTTGGGAATAACAATTTCTTCTACCTACTGCTCCACCAACCATGATTACACTATCT
GGATGCCTGAAGAACCAAGACTCGGGACATCTTGTGACATTTTTACTAATAGCAAAGGGAAAAAGGCATCTA
AGGGGGGCAAGGTTTGCGGATTTGTGGATGAAAGGGGCTTGTATAAGTCTCTAAAGGGAGCATGTAAACTCA
AGCTGTGCGGAGTTTCCGGACTTAGACTTATGGATGGAACTTGGGTTACAATTCAGACGTCAGATGAGACCAA
ATGGTGCCCTCCAGATCAATTGGTAAATCTACATGACTTTCACTCAGATGAAATAGAGCATCTCGTCGTGGAG
GAATTGGTTAAGAAGAGGGAGGAGTGTTTGGATGCATTAGAGTCCATCATGACCACCAAATCTGTAAGTTTCA

GACGTCTAAGCTATTTGAGAAAACTTGTCCCCGGGTTTGGAAAGGCATATACTATATTCAACAAGACTTTGAT
GGAAGCTGACGCCCACTACAAGTCAGTTCGGACTTGGAATGAGATCATCCCCTCAAAAGGATGTTTGAAAGTC
AGTGATAGGTGTCATCCTCATGTAGACGGAGTGTTCTTCAATGGCATAATTCTGGGTCCTGACGGGAATGTCT
TGATACCTGAAATGCAATCGTCCCTACTTCAACAACATATGGAGCTGTTGGAGTCCTCTGTAATCCCCTTGATG
CATCCCTTGGCAGACCCATCAACAGTATTCAAGGATGGAGACGAAGCGGAGGACTTTGTTGAAGTTCACCTCC
CGGATGTTCACAAACAGATCTCAGGGGTTGACCTCGGTCTCCCTAGTTGGGGGAAGTATCTCCTCATGATTGC
AGGTGGTCTAGCGACACTAGTTCTGATAATCTTCTTGATGGCATGTTGTAGAAGAACCAAGAGAACAGAATCA
AGACGGCGAGGCTCTGGGGAATCAGGAGAGAAGGTATTGGCAACTCCCCAACCCAAGAAAGTCGTATCTTCA
TGGGAGTTGTACAAGAGAGAAGGCGATGCCAGGCTGTGA

>RABV_canid variant_ 38/20_Complete glycoprotein cds_P#11

ATGGTTCCTCAGGCTCTTTTGTTTTTACCCCTTTTGGTTTTTCCATTGTGTTTCGGGAGATTCCCTATCTACACGA
TACCAGACAAACTTGGACCCTGGAGCCCAATTGACATACATCATCTCAGCTGCCCAAACAATTTGGTTGTGGA
GGACGAAGGATGCACCAACCTGTCAGGGTTCTCCTACATGGAACTCAAAGTTGGATACATCTCAGCCATAAAG
GTGAACGGATTCACTTGCACAGGCGTTGTGACTGAGGCTGAAACCTACACTAACTTTGTAGGTTATGTCACCA
CCACGTTCAAAAGAAAGCATTTCCGCCCAATACCAGACGCATGTAGAGCCGCATACAACTGGAAGATGGCCG
GTGACCCCAGATATGAAGAGTCTCTACACAATCCGTACCCTGACTACCACTGGCTTCGAACTGTAAAAACCACC
AAGGAGTCTCTCGTTATAATATCCCCAAGTGTGGCGGATTTGGACCCATATAACAAATCCCTTCACTCGAGGGT
CTTCCCCAGCGGGAAGTGCTCAGGAATAACGGTGTCTTCTACCTTCTGCTCAACTAACCACGATTACACCATTT
GGATGCCTGAAAATCCGAGACTGGGGACGTCTTGTGACATTTTCGCCAATAGTAGAGGGAAGAGAGCATCTA
AAGGGGGTAAGACCTGCGGCTTTGTAGACGAAAGAGGCCTGTATAAATCTCTAAAGGGAGCATGCAAACTCA
AGTTATGTGGAGTTCTCGGACTTAGACTCATGGATGGAACATGGGTCGCGATGCAAACATCAGATGAGACCA
AATGGTGTCCCCCTGACCAGTTGGTAAATATACACGACTTTCGCTCGGATGAAATTGAGCATCTTGTTGTGGA
GGAGTTAGTTAAGAAAAGAGAGGAGTGTCTGGATGCACTAGAGTCCATCATGACCACCAAGTCAGTGAGTTT
CAGACGTCTCAGTCACTTGAGAAAACTTGTCCCTGGGTTCGGAAAAGCATACACCATATTCAACAAGACCTTG
ATGGAGGCTGATGCTCACTACAAGTCGGTCCGGACTTGGAATGAGATCATCCCCTCAAAAGGGTGTTTAAGA
GTTGGAGGGAGGTGTCATCCCCATGTGAACGGGGTGTTCTTCAATGGTATAATATTAGGGCCCGACGGCCAT
GTTCTGATCCCCGAGATGCAATCATCCCTCCTTCAGCAACATATGGAGTTATTGGAATCCTCAGTTATTCCCCTG
ATGCACCCTCTGGCAGACCCGTCCACAGTTTTCAAGGACGGTGATGAGGCTGAGGATTTTGTAGAGGTTCACC
TCCCTGATGTGCACAAACAGGTCTCGGGGGTTGACCTAGGTCTCCCGAACTGGGGGAAATATATATTACTGAG
TGCAGGGACCCTGATTGCCTTGATGTTGATAATTTTTCTAATGACATGTTGTAGAAGAGTCAATCGACCAGAGT
CTACTGAACGCAGTCTCGGAGGGACAGGGAGGAAGGTGTCGGTCACTTCCCAAAGCGGAAAGGTCATATCTT
CATGGGAGTCATATAAAAGTGGGGGTGAGACTAGATTGTAA

>RABV_mongoose variant_114/20_Complete glycoprotein cds_P#12

ATGTCTTCTCAGTCTCTTTTGTTTGTATCCCTTCTGGGTTTTTCACTGTGTTTCGGGAAGTTCCCAATTTACACGA
TACCAGATAAACTTGGTCCCTGGAGCCCGATTGATATACATCATCTCAGTTGTCCAAACAATTTGGTAGTGGAG
GATGAAGGATGCACTAACCTATCGGGATTCTCCTACATGGAACTTAAAGTGGGATACATCTCAGCCATAAAAG
TAAATGGGTTTACGTGTACAGGTGTGGTGACGGAGGCAGAAACCTATACAAACTTTGTCGGTTATGTCACCAC
CACGTTCAGAAGAAAGCATTTCCGCCCTACACCGGATGCATGTAGATCCGCGTACAACTGGAAGATGGCCGG
TGACCCCAGATATGAAGAGTCTCTGCACAATCCATACCCTGACTACCACTGGCTTCGCACCGTCAAAACCACCA
AGGAATCTCTCGTCATAATATCCCCTAGTGTGGCAGATTTGGACCCATATGACAACTCCCTTCATTCGAGGGTC
TTCCCCAACGGAAAGTGCTCAGGGCTAACTGTATCCTCTACCTATTGCTCGACCAACCATGACTACACTATCTG
GATGCCTGAGAATCCAAGACTGGGGACATCTTGTGACATCTTTACCAATAGTAGAGGAAAGAGAGCATCCAG

AGGAAACAAGACCTGCGGATTCGTGGATGAGAGAGGCTTGTATAAGTCTCTAAAAGGAGCATGTAAACTCAA
ATTGTGTGGTGTTCTTGGACTGAGGCTTATTGATGGAACATGGGTCGCGATGCACACATCGGATGAGACCAA
ATGGTGCCCCCCcGATCAGTTGGTAAATCTACATGACTTCCGCTCAGACGAGATTGAACATCTCGTTGTGGAG
GAGTTGGTCAAGAAAAGAGAGGAGTGTTTGGATGCATTAGAATCCATCATGACCACCAAGTCAGTGAGTTTC
CGACGTCTCAGTCACTTGAGAAAACTTGTCCCCGGGTTCGGAAAAGCATATACCATCTTCAACAAAACCTTGAT
GGAGGCCGATGCTCACTACAAGTCAATCCGGACCTGGGATGAAATCGTCCCCTCGAAAGGATGTCTAAGGGT
GGGAGGGAGATGTCATCCTCATGTAAATGGGGTGTTTTTCAATGGCATAATACTGGGGTCTGACGGCCATGTT
CTAATCCCCGAGATGCAATCATCCCTCCTTCAGCAACATGTAGAGTTGCTGGAATCCTCAGTTGTTCCCTTAAT
GCACCCTTTGGCAGACCCATCTACAGTCTTTAAGGACGGAAATGAAGCTGAAGATTTTGTTGAGGTTCACCTC
CCCGATGTACACAAGCAGGTCTCAGGGGTTGACTTGGGTCTCCCAGACTGGGGAAGGTATATTCTGGTGAGT
GCAGGAGCCCTGATTACCTTGATTTTGATAATTTTCCTAATAACATGTTGCAGAAGAGTCTATCGACGAGAATC
CAAGCAACACAGTCTCAGAGGGACAGAGAGGAAGGTGTCGGTCACATCTCAAAGCGGAAAAGCCATATCCTC
ATGGGAGTCATATAAGAACGGGAGTGGAACCAGAATGTGA

>SHIBV_RV3381_Complete glycoprotein cds_P#11

ATGAGCAATTTATGCACCATTTTCATCCTGTGTGCAAGTATAATGGTTTCTTTAGGTGATTTTCCACTGTACACA
ATTCCCGAGAAAATAGGACCATGGACGCCCATTGATTTGACCCATCTGAGTTGTCCAAACAACCTGCTTTCAGA
AGATGACGGATGCAGCTCTTCCTCAACTTTCAGTTATATAGAATTGAGAACTGGTTATCTCACCCATCAAAAAG
TGTCTGGATTTACGTGTACTGGAGTCATCAACGAAGCAGTTACATACACCAATTTTGTAGGATATGTAACAACC
ACTTTTAAAAGAAAACATTTCAAACCAACTGCTTCTGCCTGTAGGGATGCATATCATTGGAAAATTTCTGGAGA
TCCTCGTTATGAAGAATCACTACACACTCCTTACCCGGACAACAGTTGGTTGAGAACAGTCACTACAACCAAA
GAATCACTTCTAATCATCTCTCCAAGCATAGTGGAGATGGACATTTATAGTCGTTCTCTTCATTCACCAATGTTT
CCCACAGGGAGGTGTTATGACTTCTATAAATCTACCCCTTCTTGCTTGACCAACCATGACTATACAATATGGCTT
CCTGATGATGCAAATGTACGACTCACATGCGATATCTTTGTAACGAGTACCGGAAAGAAATCAATGAATGGTT
CAAAAATGTGTGGCTTTACTGATGAAAGAGGACTTTATAGAACTCTGAAAGGGGCTTGTAAGTTGACATTATG
TGGAAAGCCAGGACTGAGATTGTTCGATGGCACCTGGATTTCTATCACAAGACCTGAGATTGTTATGTGGTGT
TCTCCTAATCAACTTGTGAATGTGCATAACAACAGAGTGGATGAGATAGAACACTTAATTGTGGGTGACTTGA
TCCGTAGAAGAGAAGAGTGCCTGGATACCTTAGAGACAGTTCTTATGTCAAAGTCAGTTAGTTTTAGACGACT
GAGTCATTTCAGAAAGCTTGTCCCGGGTTTTGGAAAGGCATATACCATCGCCAATGGGAGCCTAATGGAAACC
AATGTACACTACAAAAGAGTAGATCGTTGGGAGGAAATATTACCCTCCAAAGGGTGCCTAAAACTGAATGAC
AAGTGTCTTAACCCTGAGAATGGGGTGTTTTTAATGGGATCATTAAAGGCCCTGATGGACAGGTTTTAATTC
CAGAAATGCAGTCAAGTTTATTGAAGCAACATATGGACTTGCTGAAAGCTTCTGTATTTCCTCTAAGACATCCT
TtGATCGATCAaAcaTCTATCTTCAAAAAAGATGGAGAGGCAGATGACTTTGTTGATGTTCACATGCCAGATCC
GcATAAGTCAATaTcCaATATAGATTTAGGTTTACCAGACTGGGGTCTATATGCAATGATTGGGGGAACTGTAG
TTGCATTTCTAATATTGGTGTGTCTCCTCTGTACTTGTTGTAAAAGAAGACGAAGGAGGAATTCCAGAAAGCCT
AGCTCAGAACAAACCCCAAAAATTTCATCTACTCCTCCTTCTGGGACTAAAGTCATATCATCTTGGGAGTCTTAT
AAAGGCACATCTAGTGTGTAA

>WCBV_RV3384_Complete glycoprotein cds_P#7

ATGGCTTCCTACTTTGCGTTGGTCTTGAACGGGATCTCTATGGTTTTCAGTCAAGGTCTTTTCCCCCTTTACACT
ATCCCTGACCATCTGGGACCATGGACCCCCATAGATCTAAGTCACCTTCACTGCCCGAACAATCTTTATACTGA
TGCCTCTTATTGTACAACTGAACAAAGCATAACCTACACAGAGTTGAAGGTCGGATCATCTGTGTCACAAAAA
ATCCCCGGATTTACATGTACGGGGGTAAGAACTGAATCTGTAACATATACCAACTTTGTTGGCTATGTGACTAC
CACGTTCAAGAAAAAACACTTTCCTCCTAAATCCAGGGACTGTAGAGAGGCGTATGAGAGGAAGAAAGCAGG

AGATCCTAGATATGAAGAGTCTTTAGCCCACCCATATCCTGACAACAGTTGGCTGAGAACAGTGACTACAACA
AAGGATTCCTGGGTGATCATCGAGCCCAGTGTAGTGGAGTTAGATATATACACAAGTGCCTTGTATTCACCTC
TTTTCAAGGATGGAACATGTTCAAAATCTAGAACATATTCCCCCTACTGTCCAACCAATCATGACTTCACCATTT
GGATGCCAGAGAGTGAAAACATAAGATCTGCCTGTAATCTGTTTTCCACAAGTAGAGGGAAACTAGTCAGGA
ACCGCACATCCACCTGCGGGATTATCGATGAGAGAGGGCTGTTCAGATCAGTTAAAGGAGCATGCAAAATAT
CAATATGCGGTAGGCAGGGAATCCGTTTAGTGGATGGAACTTGGATGTCTTTTAGATACTCAGAGTACTTACC
TGTGTGTTCTCCATCACAGCTGATCAACACGCACGACATCAAGGTCGATGAGCTGGAGAATGCTATAGTTTTA
GACTTGATTAGGAGGAGAGAAGAATGTCTTGACACCCTAGAAACAATTTTGATGTCAGGATCTGTGAGTCACA
GGAGGCTGAGTCATTTCAGAAAGCTGGTTCCAGGATCTGGGAAGGCTTACTCTTATATAAACGGCACCTTAAT
GGAATCAGATGCTCACTACATCAAGGTAGAGAATTGGTCAGAGGTCATCCCACACAAAGGATGTCTCATGGTC
GGGGGCAAATGCTATGAGCCAGTCAATGATGTGTATTTCAACGGGATCATTCGGGATTCAAATAATCAGATCT
TGATACCTGAGATGCAGTCCAGTCTTCTCAGAGAACATGTTGACCTGTTGAAGGCTAATATAGTTCCGTTCAG
GCATCCAATGTTACTTAGGTCCTTCACATCTGACACTGAAGAAGATATCGTCGAGTTTGTCAACCCTCATCTCC
AAGATACCCAGAAGTTGGTGTCAGATATGGATCTCGGGTTATCAGACTGGAAGAGATATCTACTAATTGGATC
TTTGGCCGTAGGAGGAGTGGTAGCAATCTTATTCATCGGAACATGTTGTCTGAGATGTAGAGCAGGGAGAAA
CAGAAGAACAATCCGATCCAATCATAGGTCATTGTCCCATGACGTGGTGTTCCATAAAGATAAGGATAAAGTG
ATTACTTCTTGGGAATCTTACAAGGGACAAACTGCCCAATAA
